# A simulated annealing algorithm for randomizing weighted networks

**DOI:** 10.1038/s43588-024-00735-z

**Published:** 2024-12-10

**Authors:** Filip Milisav, Vincent Bazinet, Richard F. Betzel, Bratislav Misic

**Affiliations:** 1 Montréal Neurological Institute, McGill University, Montreal, Quebec Canada; 2 Department of Neuroscience, University of Minnesota, Minneapolis, MN USA

**Keywords:** Network models, Complex networks, Computational neuroscience

## Abstract

Scientific discovery in connectomics relies on network null models. The prominence of network features is conventionally evaluated against null distributions estimated using randomized networks. Modern imaging technologies provide an increasingly rich array of biologically meaningful edge weights. Despite the prevalence of weighted graph analysis in connectomics, randomization models that only preserve binary node degree remain most widely used. Here we propose a simulated annealing procedure for generating randomized networks that preserve weighted degree (strength) sequences. We show that the procedure outperforms other rewiring algorithms and generalizes to multiple network formats, including directed and signed networks, as well as diverse real-world networks. Throughout, we use morphospace representation to assess the sampling behavior of the algorithm and the variability of the resulting ensemble. Finally, we show that accurate strength preservation yields different inferences about brain network organization. Collectively, this work provides a simple but powerful method to analyze richly detailed next-generation connectomics datasets.

## Abstract (web summary)

This study proposes an algorithm for generating randomized networks that preserve the weighted degree sequence. The procedure outperforms standard rewiring algorithms and extends to multiple network types, including directed and signed networks.

## Main

The connectome is a complex network that constitutes a comprehensive catalog of the brain’s neural elements and their connections^1^. Numerous topological features of structural brain networks have been identified, including high clustering, short characteristic path length^2–6^ and a rich-club of highly interconnected hub nodes^7–10^. These network features are consistently expressed across species^11^, spatial scales, neuroimaging modalities and tract-tracing technologies^12^.

To quantify the unexpectedness of brain network features, network-based statistics are typically compared against null features computed in populations of randomized networks that preserve specific attributes of the empirical network^13^. Comparisons with randomized networks can then be used either for statistical inference or for benchmarking. In the case of statistical inference, a *P* value for a particular network feature can be estimated by computing the proportion of randomized networks for which that feature has a more extreme magnitude than in the empirical network. In the case of benchmarking, a network feature can be normalized against the distribution of that feature in the population of randomized networks. For instance, the oft-studied small-world coefficient^2,14,15^ and the rich-club coefficient^16,17^ are by definition normalized in such a manner. The most widely used network null model is degree-preserving rewiring (often referred to as Maslov–Sneppen rewiring^18^), which uses edge swapping to disrupt the empirical network’s topology but preserves its size (that is, number of nodes), density (that is, proportion of expressed edges) and binary degree sequence (that is, number of edges incident to each node). Importantly, by selectively controlling for lower-order features, null models can be used to rule out the possibility that higher-order structures reflect random assemblies of simpler features^13^.

However, with the advent of rich weighted networks—spanning up to six orders of weight magnitude^19–21^—and their increasing use over their simpler binary counterparts, there is a need for null models that accommodate weighted network statistics. For example, in diffusion-weighted magnetic resonance imaging (MRI) tractometry, edge weights are conventionally estimated using numbers of streamlines or fractional anisotropy^22^. More recently, numerous metrics have been developed to more directly quantify microstructural attributes of network edges^23^, including neurite density^24^, myelin^25–27^, axon diameter^28,29^ and axon cross-sectional area^30,31^. Invasive methods in animal models also yield weighted networks, including tract tracing using fluorescent markers^32–34^, genetic labeling^35^ and electron microscopy^36,37^. Additionally, brain networks are increasingly reconstructed by means of comparing inter-regional similarity^38,39^, such as gene coexpression^40,41^, laminar profile covariance^42^ or receptor similarity^43^, again yielding biologically meaningful edge weight distributions. Therefore, next-generation connectomics requires new randomization algorithms that take edge weights into account.

Several network null models have been developed that preserve the weighted degree sequence (hereafter referred to as strength sequence) of empirical networks in addition to their binary degree sequence^17,44–50^. Most of these models are sampling methods based on maximum likelihood estimation of a network probability distribution^44,45,51^. Here, we do not consider these models for two reasons. First, they only satisfy the degree sequence constraints on average across the complete network ensemble but not for each individual sampled network^52,53^. Second, these models do not maintain the empirical network’s weight distribution^53^. Furthermore, for these reasons, they are seldom practically used in network neuroscience.

Here, we present an algorithm that addresses these limitations by preserving an empirical network’s weight distribution, degree sequence and strength sequence for each randomized network instance. Contrary to other strength-preserving models, this procedure does not require any analytical derivations, instead building on classic rewiring techniques already commonplace in the field of network neuroscience and network science more generally. This randomization technique reconfigures weight placement atop the binary scaffolding of a rewired network to match the empirical network’s strength sequence using simulated annealing, a probabilistic algorithm that approximates the global minimum of a given function^54,55^. Simulated annealing is a powerful and versatile optimization technique with wide-ranging applications. Moreover, it is particularly advantageous when dealing with large combinatorial search spaces, making it a prime candidate for solving network modeling problems^6,52,53,56–64^.

In this study, we benchmark the performance of the simulated annealing procedure against another rewiring algorithm for strength sequence-preserving randomization (hereafter referred to as the Rubinov–Sporns algorithm^65^), as well as the classic Maslov–Sneppen degree-preserving rewiring model^18^. In parallel, we use morphospace representation to assess null network variability, a seldom considered^46,61,66–68^ but important evaluation step when comparing network null models.

## Results

### Model overview

**Fig. 1:**
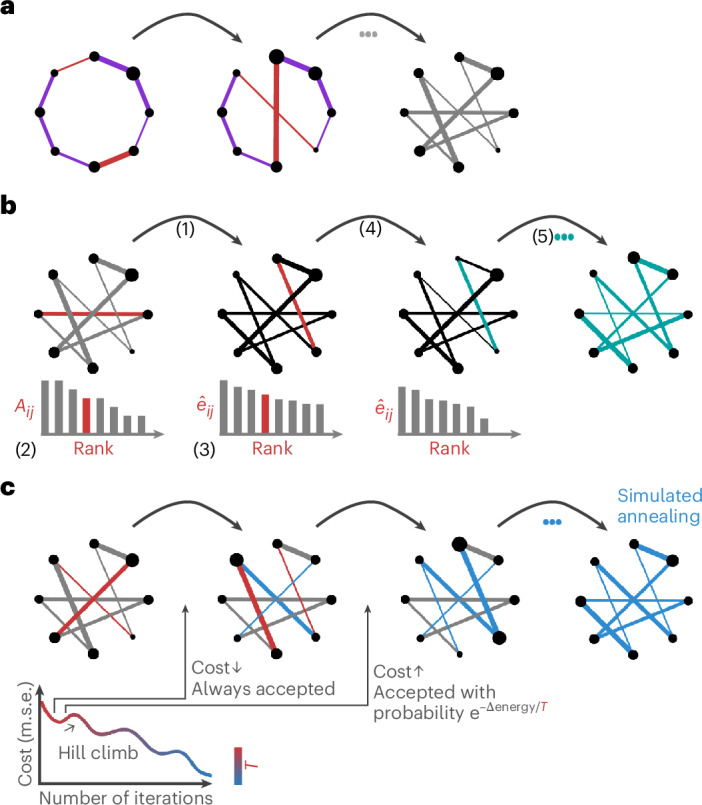
Rewiring algorithms for generating strength sequence-preserving randomized null networks. **a**, Maslov–Sneppen degree-preserving rewiring^18^. Pairs of edges (red) are randomly swapped, disrupting the network’s topology but preserving its size, density and degree sequence. Edge width represents weight, and node size represents strength. **b**, Rubinov–Sporns strength sequence-preserving randomization algorithm^65^. Using the Maslov–Sneppen rewired network: (1) the randomized network is instantiated with zeros (${\hat{A}}_{ij}=0$, black), (2) the original edge weights (*A*_*ij*_) are ranked by magnitude (left), (3) the edges of the randomized network are ranked by their expected magnitude (${\hat{e}}_{ij}$, middle), (4) a random edge is selected in the randomized network and its weight is set to the original edge weight of the same rank (both edges are depicted in red) and (5) the edges are reranked, and the procedure is repeated, resulting in the Maslov–Sneppen rewired network, with edge weights permuted to approximate the empirical network’s strength sequence (right). ${\hat{e}}_{ij}\propto ({s}_{i}-{\Sigma }_{u}{\hat{A}}_{iu})({s}_{j}-{\Sigma }_{u}{\hat{A}}_{ju})$, where *s*_*i*_ represents the strength of node *i* in the empirical network and ${\Sigma }_{u}{\hat{A}}_{iu}$ is the sum of the weights of assigned edges incident to node *i* in the randomized network. In the middle networks, edge width represents expected weight magnitude (black or red) or assigned weight (teal) and node size represents residual strength (${s}_{i}-{\Sigma }_{u}{\hat{A}}_{iu}$). **c**, Strength sequence-preserving randomization via simulated annealing. Using the Maslov–Sneppen rewired network, randomly selected pairs of edge weights (red) are permuted either if they lower the energy of the system (mean squared error (m.s.e.) between the strength sequences of the empirical and the randomized networks) or if they meet the probabilistic Metropolis acceptance criterion, depending on the temperature (*T*) of the system.

Briefly, we consider three rewiring algorithms (Fig. 1; see Methods for a detailed description). In the classic Maslov–Sneppen algorithm, pairs of edges are randomly swapped and weights are ‘carried’ with their respective edges^18^ (Fig. 1a). The Rubinov–Sporns algorithm, building on the output of the Maslov–Sneppen algorithm, attempts to preserve the strength sequence by sampling each edge weight, in pseudorandom order, from the original edge weight distribution using a rank-matching procedure^65^ (Fig. 1b). Finally, in the simulated annealing procedure, also building on the output of the Maslov–Sneppen algorithm, randomly selected pairs of edge weights are permuted either if they lower the energy of the system (mean squared error between the strength sequences of the empirical and the randomized networks) or if they meet a probabilistic acceptance criterion. This allows permutations which can increase the energy of the system but prevents it from getting stuck in a local minimum (Fig. 1c). Importantly, the procedure is also applied on the Maslov–Sneppen rewired network.

The experiments are performed in two publicly available diffusion-weighted MRI datasets, acquired using different protocols (diffusion spectrum imaging (DSI) and high angular resolution diffusion imaging), parcellations (anatomical and functional) and parcellation resolutions (low and high in each dataset). The first sample (LAU) consists of DSI data acquired in *n* = 70 participants (Lausanne University Hospital^69^; see Methods for detailed procedures). The second sample consists of high angular resolution diffusion imaging data acquired in *n* = 327 participants (Human Connectome Project (HCP)^70^, see Methods for detailed procedures). For both datasets, group-representative weighted structural networks were built using a distance-dependent consensus-based thresholding procedure^71,72^, resulting in a total of four empirical group-consensus networks (LAU, low resolution; LAU, high resolution; HCP, low resolution; HCP, high resolution) on which the main analyses were conducted (see ‘Strength-preserving randomization in individual networks’ section for an analysis of individual participant connectomes). Here, the results are visualized in the high-resolution HCP dataset, but exhaustive figures are provided as Supplementary Information (Supplementary Figs. 7, 8, 10, 12 and 14).

### Benchmarking strength sequence preservation

**Fig. 2:**
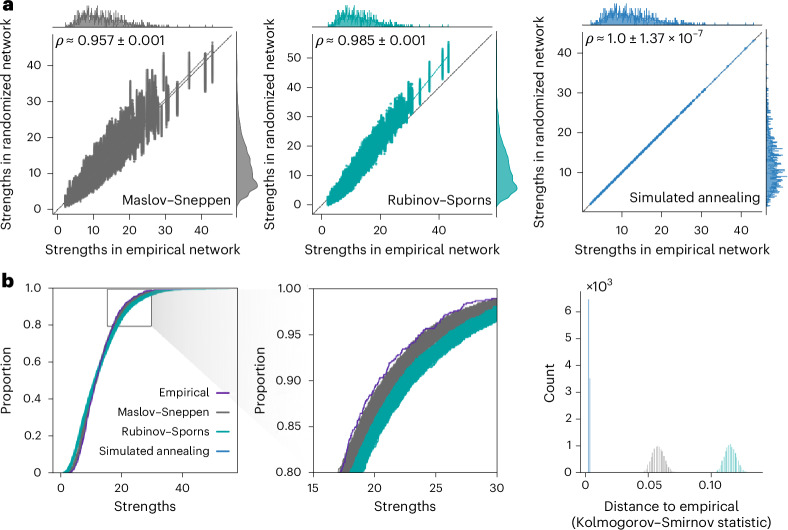
Benchmarking strength preservation. **a**, Scatter plots of strengths of the empirical (abscissa) and randomized (ordinate) networks for all 10,000 null networks, where each point represents a brain region. The marginal distribution histograms are shown on the top and right axes. The mean and standard deviation across 10,000 Spearman rank-order correlation coefficients are given in the top left of each panel. The data points and histograms appear in gray for the Maslov–Sneppen algorithm, teal for the Rubinov–Sporns algorithm and blue for the simulated annealing algorithm. Linear regression lines (colored) are computed over the whole ensemble for visualization purposes. The identity line (black) is provided as a reference. **b**, Strength cumulative distribution functions (left) and histograms representing Kolmogorov–Smirnov statistics obtained by comparing the strength distribution of the empirical network with that of the randomized networks (right). Cumulative distribution function curves and histograms are shown in gray for the Maslov–Sneppen algorithm, teal for the Rubinov–Sporns algorithm and blue for the simulated annealing algorithm. The original cumulative distribution function is depicted in indigo and almost perfectly overlays all 10,000 cumulative distribution functions obtained via simulated annealing, effectively hiding them. Middle: a close-up of the rectangle on the left. Source data

We benchmark the performance of the randomization algorithms by generating 10,000 null networks for each empirical brain network. To characterize the performance of the null models in preserving strength sequence—the ordered set of strengths in which each node is associated to a specific strength—we plot empirical network strengths against strengths of the randomized networks for all 10,000 nulls (Fig. 2a and Supplementary Fig. 7). We also compute Spearman rank-order correlation coefficients between empirical and ‘randomized’ strengths. Across all four empirical brain networks, the simulated annealing algorithm yields near perfect fits (LAU, low resolution: mean = 0.999, standard deviation = 0.001; LAU, high resolution: mean = 0.996, standard deviation = 0.002; HCP, low resolution: mean ≈ 1.0, standard deviation = 3.04 × 10^−7^; HCP, high resolution: mean ≈ 1.0, standard deviation = 1.37 × 10^−7^). It also results in larger correlation coefficients than the Rubinov–Sporns algorithm (*P* ≈ 0, common-language effect size (CLES) of 100% for all empirical networks, two-tailed, Wilcoxon–Mann–Whitney two-sample rank-sum test), which itself results in larger coefficients than the Maslov–Sneppen algorithm (*P* ≈ 0, CLES of 100% for all empirical networks, two-tailed, Wilcoxon–Mann–Whitney two-sample rank-sum test). This shows that the simulated annealing algorithm generates randomized networks with the most veridical strength sequences. In Supplementary Information, we further investigate null model calibration (‘Null model calibration’ section) and provide sensitivity analyses examining the effect of an alternative objective function (‘Alternative objective function’ section), annealing schedule (‘Alternative annealing schedule’ section) and weight log transformation on simulated annealing performance (‘Log-transformation’ section). We also provide an in-depth analysis of the model’s computational cost, characterizing its tradeoff with performance and scaling with network density and size (‘Computational cost’ section).

### Benchmarking strength distribution preservation

While we have established, using rank-based methods, that the simulated annealing algorithm outperforms other randomization techniques in preserving the empirical network’s strength sequence, we have not quantified how well the different models preserve the strength distribution. The level to which the empirical strength distribution is preserved in a null network is crucial, because it ensures an accurate representation of influential graph features, such as hubs, whose importance is intricately tied to characteristics of the distribution.

To assess the goodness of fit between the strength distributions of the empirical and the randomized structural networks, we superimpose their cumulative distribution functions (Fig. 2b and Supplementary Fig. 8). Across all datasets, the curves produced via simulated annealing show the best match to the empirical strength cumulative distribution function with almost perfect superposition. Furthermore, the curves obtained using the Rubinov–Sporns and the Maslov–Sneppen algorithms show considerably more variability across null networks as shown by their wider spread, recapitulating previously observed patterns of underestimation and overestimation across datasets (see ‘Null model calibration’ section in Supplementary Information). To confirm these observations quantitatively, we compute Kolmogorov–Smirnov test statistics between the cumulative strength distributions of the empirical and each randomized network, measuring the maximum distance between them (Fig. 2b and Supplementary Fig. 8). Across all datasets, the simulated annealing algorithm outperforms the other two null models with significantly lower Kolmogorov–Smirnov statistics (*P* ≈ 0, CLES of 100% for all two-tailed, Wilcoxon–Mann–Whitney two-sample rank-sum tests). Furthermore, in the HCP dataset and the higher resolution Lausanne network, the Rubinov–Sporns algorithm generated cumulative strength distributions with slightly worse correspondence to the empirical distribution than the cumulative strength distributions yielded by the Maslov–Sneppen algorithm (LAU, high resolution: *P* < 10^−176^, CLES of 61.58%; HCP: *P* ≈ 0, CLES of 100% for all empirical networks, two-tailed, Wilcoxon–Mann–Whitney two-sample rank-sum test).

As an illustration, we consider whether the nulls generated by different algorithms recapitulate fundamental characteristics associated with the empirical strength distribution. Namely, we focus on the heavy tailedness of the strength distribution (that is, does the null network also have a heavy-tailed strength distribution, suggesting the presence of hubs?) and the spatial location of high-strength hub nodes. We assess heavy tailedness and identify hubs using the nonparametric procedure outlined in refs. ^73,74^ (see Methods for more details).

Considering the high-resolution Lausanne dataset as an example, we find that the empirical group-consensus network has a heavy-tailed strength distribution, with 2% of the nodes identified as hubs (Supplementary Fig. 9); Supplementary Fig. 9 shows their spatial location in red. By comparison, across 10,000 realizations, the Maslov–Sneppen algorithm only recapitulates heavy tailedness 0.03% of the time, and the spatial locations of hubs do not recapitulate the empirical map. The Rubinov–Sporns algorithm does better and detects heavy tailedness in all realizations but identifies only 1.4% of nodes as hubs on average (compared with 2% in the empirical network). Finally, the simulated annealing algorithm detects heavy tailedness in all realizations and identifies 1.96% of nodes as hubs on average, providing the closest match to the empirical network. We also assess how well each algorithm recovers the correct spatial location of these hubs using *z*-scored Rand indices between the empirical and the null hub assignments and find that simulated annealing also outperforms the other algorithms in recapitulating hub identity (*P* ≈ 0, CLES of ≈ 100% for both two-tailed, Wilcoxon–Mann–Whitney two-sample rank-sum tests) (Supplementary Fig. 9).

### Null network ensemble variability

**Fig. 3:**
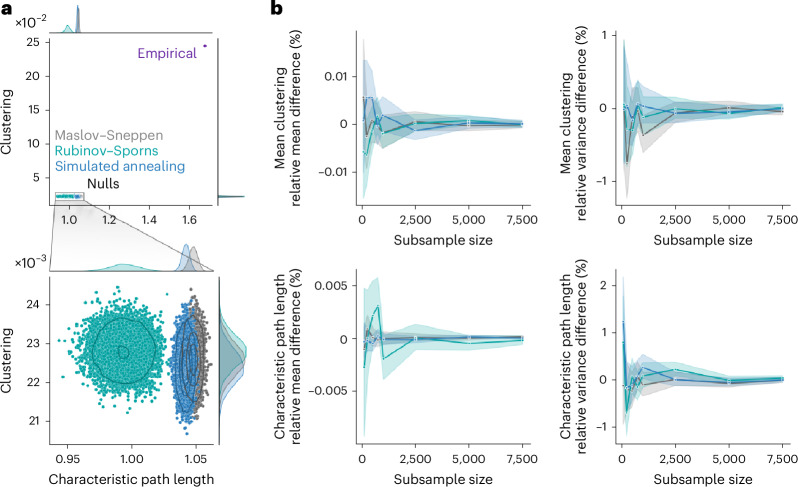
Morphospace of null network ensembles. **a**, Morphospaces spanned by characteristic path length and clustering, with data points corresponding to randomized null networks generated by the Maslov–Sneppen (gray), Rubinov–Sporns (teal) and simulated annealing (blue) algorithms, plus the empirical group-consensus structural network (indigo). Bottom: a zoomed-in view of the clusters of randomized networks appearing in the top. The marginal distribution histograms are shown above and to the right of the plots. The contour levels are drawn using a Gaussian kernel density estimate and delineate isoproportions of the density. **b**, The trajectories of relative difference in mean clustering (top left), clustering variance (top right), mean characteristic path length (bottom left) and characteristic path length variance (bottom right) between the full null population (*N* = 10,000) and subsamples of increasing size (*n* ∈ {100, 250, 500, 750, 1,000, 2,500, 5,000, 7,500}). The colored lines and shaded bands represent the mean and a 95% bootstrapped confidence interval (1,000 samples), respectively. As in **a**, colors represent the randomization algorithm used. Source data

Generating rewired null networks for the purpose of null hypothesis testing or normalization is ubiquitous in network neuroscience and in network science more generally^13^. Yet the features derived from ensembles of rewired nulls are typically averaged or summarized, without considering the variability across network realizations. Whether all randomization algorithms produce ensembles of null networks that vary to the same extent is unknown. Do different algorithms yield null networks with comparable architectural features? Do different algorithms sample the space of possible null networks differently? To assess variability of network surrogate realizations, we embedded the 10,000 nulls generated for each randomization algorithm and each of the four empirical networks in a two-dimensional morphospace spanned by two global weighted network statistics: characteristic path length and clustering^75–79^ (Fig. 3a). Note that this method could have been applied with any other network statistic; we chose characteristic path length and clustering because of their wide use in the network science literature, notably as a means to study small-worldness^2–6^.

Across datasets, we observe that all randomized networks generally occupy the same portion of the morphospace relative to the empirical networks (Fig. 3a and Supplementary Fig. 10). This indicates that, despite the further constraints in edge weight placement imposed upon the strength sequence-preserving algorithms, they produce patterns of global weighted network features similar to those of the classic Maslov–Sneppen degree-preserving rewiring. While this is true when comparing the position of randomized networks to that of the empirical network, zooming in on the region of the morphospace occupied by the null networks (Fig. 3a and Supplementary Fig. 10) reveals important differences in their variability.

Null networks derived using simulated annealing organized in patterns similar to those of the Maslov–Sneppen rewired networks. Namely, the simulated annealing ensemble retains a similar shape, similar density distribution (as shown by the contour lines) and often occupies a position close to the Maslov–Sneppen ensemble that remains consistent across datasets (Fig. 3a and Supplementary Fig. 10). By comparison, the Rubinov–Sporns ensemble is less consistent across datasets and in the HCP dataset yields large density distributions in which many realizations are disproportionately concentrated in the middle. Interestingly, simulated annealing ensembles generally show similar clustering but slightly lower characteristic path length than their strictly degree-preserving counterpart (*P* ≈ 0 for all empirical networks, two-tailed, Wilcoxon–Mann–Whitney two-sample rank-sum test; LAU, low resolution: CLES of 97.66%; LAU, high resolution: CLES of 100%; HCP, low resolution: CLES of 89.11%; HCP, high resolution: CLES of 90.26%). While this shows that the simulated annealing algorithm yields networks that are more dissimilar to the empirical network in terms of their characteristic path length as compared with Maslov–Sneppen rewiring, it also identifies them as slightly more stringent benchmarks when assessing how unexpectedly low an empirical network’s characteristic path length is, such as when computing the small-world coefficient^2,14^.

Another question that naturally emerges when using network null models is how many nulls to generate. While the scaling behavior of a network feature’s null distribution probably varies depending on the feature at hand, morphospaces might provide insight into the question by summarizing global aspects of a network’s architecture. In Supplementary Fig. 11, we consider a subsample of 100 nulls out of the 10,000 generated. We see that the same patterns of null network ensemble organization already seem to emerge with only 100 nulls, sampling a similar extent of the morphospace. To quantify the scaling behavior of the morphospace, we consider a range of increasing subsample sizes (*n* ∈ {100, 250, 500, 750, 1,000, 2,500, 5,000, 7,500}). For each randomization algorithm and subsample size, we draw 1,000 random samples and compute the relative difference between each sample’s global network statistics (mean and variance across nulls of mean clustering and characteristic path length) and the statistics obtained in the full ensemble of 10,000 nulls. In Fig. 3b and Supplementary Fig. 12, we show the scaling behavior of the morphospace’s global network statistics as a function of null sample size. Interestingly, we find that even at the lowest sample size, relative differences do not exceed 2% for any of the statistics. Furthermore, relative differences rapidly converge to even lower values with increasing sample size. Therefore, we find that only a small number of nulls is necessary to adequately approximate the null distribution of these global network features. Altogether, these results show that explicitly considering how the space of possible null realizations is sampled is important yet often overlooked. Additionally, in Supplementary Information, we analyze morphospace trajectories, relating energy and morphospace position throughout the simulated annealing procedure (‘Morphospace trajectories’ section).

### The weighted rich-club phenomenon

To illustrate how the choice of a network null model can have important ramifications for network inference, we consider the weighted rich-club phenomenon in connectomics. A rich club is characterized as a group of high-degree nodes (rich nodes) that exhibit a greater number of interconnections than would be anticipated by chance^16,80^. In this study, we go beyond the conventional definition of rich club and incorporate a weighted measure to assess the relative strength of connections among rich nodes, referred to as the weighted rich-club coefficient^17,81^. This measure is evaluated at various threshold degree values, used to define the rich nodes (see Methods for more details). Considering that high-degree nodes are more likely to be interconnected, the (weighted) rich-club coefficient is commonly normalized against a null rich-club coefficient averaged across an ensemble of randomized null networks. Although the conventional Maslov–Sneppen degree-preserving rewiring method is frequently employed in generating the null network population, it does not factor in the effect of the weighted degree sequence in the computation of the weighted rich-club coefficient. Here, to contrast inferences obtained using different null models, we compute the weighted rich-club coefficient in each of the 10,000 null networks generated for each model under study. We then compute the normalized weighted rich-club coefficient using the average coefficient across nulls for each model and assess significance by deriving a *P* value as the proportion of null coefficients that are greater than the empirical coefficient.

**Fig. 4:**
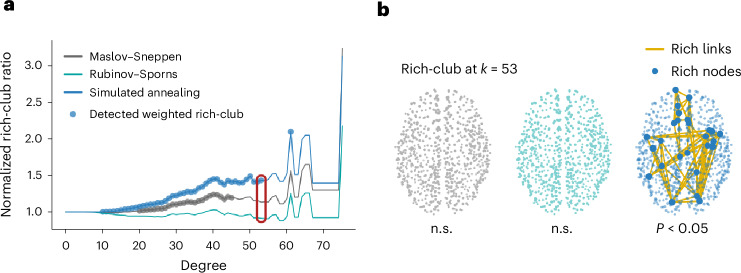
The weighted rich-club phenomenon. **a**, The normalized rich-club ratio, computed using the Maslov–Sneppen (gray), Rubinov–Sporns (teal) and simulated annealing (blue) randomization algorithms, as a function of the degree threshold used to define rich nodes. The colored points indicate significance at the Bonferroni-corrected threshold of *P* < 0.05 (one-sided), indicating that a weighted rich-club was detected. **b**, Example empirical weighted rich-club detected at *k* = 53, with *k* corresponding to degree. Node size is proportional to strength. Only the simulated annealing algorithm detects a significant weighted rich-club at *k* = 53. The rich nodes are enlarged to showcase their spatial location. n.s., not significant. Source data

Interestingly, we find that, for all empirical networks, using simulated annealing-derived null networks yields larger normalized rich-club ratios than using Rubinov–Sporns or Maslov–Sneppen randomization (*P* < 0.01 for all two-tailed, Wilcoxon–Mann–Whitney two-sample rank-sum tests) (Fig. 4 and Supplementary Fig. 14). Similar results were obtained for an alternative definition of the weighted rich-club coefficient^9,17^ (see Methods and Supplementary Fig. 15 for more details). Importantly, the Rubinov–Sporns algorithm never identifies a weighted rich-club and only the simulated annealing algorithm detects a significant weighted rich-club in the Lausanne dataset. This result might seem counterintuitive, given that null networks that embody more aspects of the empirical network are generally seen as more conservative. However, this is not a general rule, and a specific result needs to be interpreted against the backdrop of the specific analysis and null constraints at hand. Here, we posit that the differences in normalized rich-club coefficient observed between models is due to an overestimation of strength in high-degree nodes. We further suggest that this difference between empirical strength and strength expected based on degree might be due to high-degree (rich-club) nodes being interconnected by a preponderance of low-weight long-range connections^10^. In Supplementary Information, we verify these hypotheses, relating the choice of network null model to weighted rich-club inference and the spatial embedding of rich connections (‘Weighted rich-club inference and geometry’ section). Altogether, these results show how contrasting nulls embodying hierarchical constraints can provide layered insights into brain network organization. More broadly, the choice of network null model can yield fundamentally different inferences about hitherto established phenomena.

### Strength-preserving randomization in individual networks

So far, we have only used empirical networks derived from a collation of individual-specific data. Therefore, how the use of the randomization algorithms under study translates to participant-level designs remains unknown. Notably, how does empirical variability compare with null variability, and how much interindividual variability is preserved in null networks?

To address these questions, we consider 69 density-matched individual structural connectivity networks from the low-resolution Lausanne dataset. We then generate 100 null networks per algorithm and empirical network and benchmark strength-preservation performance across randomization algorithms but now in each individual network. We use an energy threshold for the simulated annealing algorithm to ensure a similar quality of strength reconstruction across networks (see Methods for more details). Supplementary Fig. 18a shows the distributions of Spearman correlation coefficient between strength sequences in empirical and randomized networks. Again, we find that the simulated annealing algorithm outperforms other null models in preserving the strength sequence (*P* ≈ 0 for both two-tailed, Wilcoxon–Mann–Whitney two-sample rank-sum tests; simulated annealing versus Rubinov–Sporns: CLES of 92.57%; simulated annealing versus Maslov–Sneppen: CLES of 100%).

Next, to contrast empirical and null variability, we embed both empirical and null networks in the same morphospace (Supplementary Fig. 18b). We observe that the empirical networks span a larger portion of the morphospace than the null networks, especially in terms of clustering. This might seem counterintuitive given the pervasive idea that null brain networks are generically ‘random’ and that, as such, they should account for a larger space of possible realizations than empirical brain networks. However, random networks constitute a class of networks in their own right with their own characteristic features, such as low clustering and short characteristic path length^2,82^, and therefore, we should not expect them to show more variability than empirical networks when assessed on that basis. Furthermore, random networks are also defined on the basis of specific structural constraints (size, density and weight distribution and degree/strength sequence in this case).

To further explore the question of interindividual variability, we focus on the null network ensembles. In Supplementary Fig. 18, we color nulls by the algorithm used to generate them (Supplementary Fig. 18d) and the empirical networks from which they are built (Supplementary Fig. 18e). We observe that, despite a considerably reduced variability, participant identity seems to dominate the organization of null networks, mimicking the empirical pattern of interindividual variability. To confirm this intuition, we measure interindividual Euclidean distance between participants in the morphospace for the empirical and null networks. We then compare algorithm-wise distributions of Spearman correlation coefficients between empirical and null Euclidean distances (Supplementary Fig. 18c). We find that simulated annealing provides a better preservation of interindividual morphospace distances than both the Rubinov–Sporns (*P* < 10^−9^, CLES of 75.23%, two-tailed, Wilcoxon–Mann–Whitney two-sample rank-sum tests) and Maslov–Sneppen (*P* < 10^−10^, CLES of 77.03%, two-tailed, Wilcoxon–Mann–Whitney two-sample rank-sum tests) algorithms. This indicates that preserving strength in addition to degree also improves the preservation of interindividual patterns of global weighted network features. In other words, weight assignment alone (or the weighted ‘hubness’ of brain regions) contributes in encoding interindividual differences.

### Strength-preserving randomization for directed networks

An advantage of randomizing networks using simulated annealing is that the optimization algorithm can be readily applied to preserve multiple network features. A straightforward extension is the preservation of in- and out-strength sequences in directed networks^53^. This is important because axonal projections are fundamentally directed, and numerous techniques can be used to reconstruct afferent and efferent connections, such as tract tracing and genetic labeling^83–86^. Here, we simply reformulate the objective function to account for the reconstruction error associated separately with in-strength and out-strength, allowing us to effectively recapitulate in- and out-strength sequences.

**Fig. 5:**
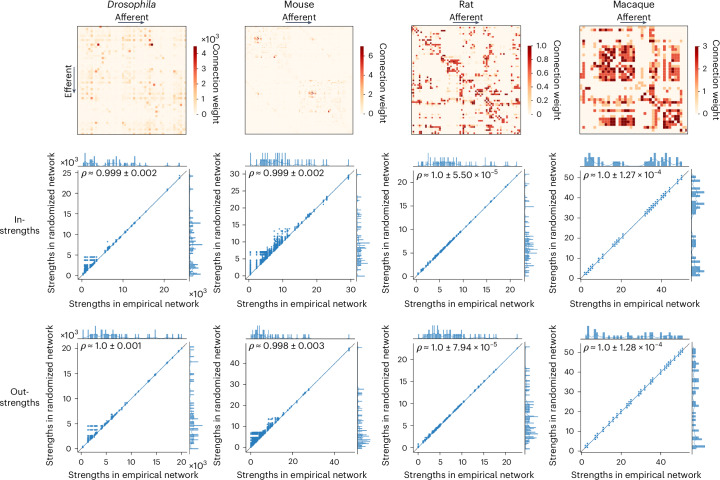
Strength-preserving randomization for directed networks. Top: wiring diagrams for the *Drosophila*, mouse, rat and macaque connectomes. Center and bottom: scatter plots of strengths of the empirical (abscissa) and simulated annealing-derived networks (ordinate) for all 10,000 nulls, where each point represents a brain region, for in-strengths (center row) and out-strengths (bottom row). Marginal distribution histograms are shown on the top and right axes. The mean and standard deviation across 10,000 Spearman rank-order correlation coefficients are given in the top left of each plot. The linear regression lines (blue) are computed over the whole ensemble for visualization purposes. The identity line (black) is provided as reference.

Figure 5 shows the directed, weighted wiring diagrams of the *Drosophila* (fruit fly)^35,87,88^, mouse^34,53^, rat^89^ and macaque^90^, along with scatter plots of strengths in the empirical and the randomized networks, separately for in- and out-strengths. Similar to the results observed in the Lausanne dataset (Supplementary Fig. 7 and Supplementary Information sections ‘Null model calibration’ and ‘Log-transformation’), we observe some low-strength inaccuracies in the *Drosophila* and mouse connectomes, which might be due to their heavily right-skewed weight distribution. However, they only have a minimal effect on strength reconstruction, as shown by the consistently high correlation coefficients obtained in all networks.

Again, we benchmark the performance of the simulated annealing algorithm against that of the Maslov–Sneppen and Rubinov–Sporns algorithms (Supplementary Fig. 16). We find that simulated annealing’s in-strength sequence reconstruction surpasses that of both the Maslov–Sneppen and Rubinov–Sporns algorithms (*P* ≈ 0, CLES of [98.65, 100]% for all networks, two-tailed, Wilcoxon–Mann–Whitney two-sample rank-sum test). In contrast, its out-strength reconstruction is more accurate than that of the Rubinov–Sporns algorithm (*P* ≈ 0, CLES of [98.74, 100]% for all networks, two-tailed, Wilcoxon–Mann–Whitney two-sample rank-sum test) but does not surpass the accuracy of the Maslov–Sneppen algorithm, which perfectly preserves out-strengths by design. To take advantage of this built-in advantage of the Maslov–Sneppen algorithm, we additionally provide an alternative version of simulated annealing for directed networks that also perfectly preserves out-strengths (‘Alternative strength-preserving randomization for directed networks’ section in Supplementary Information). Overall, these results demonstrate how the procedure can be readily adapted to accommodate additional network formats (see ‘Strength-preserving randomization for signed networks’ section in Supplementary Information for how simulated annealing can be adapted for signed networks).

### Strength-preserving randomization across network classes

Until now, we have only considered networks that represent inter-regional connectivity in brains. Yet the simulated annealing algorithm presented here is generic and can be readily applied to other classes of complex networks. For completeness, we benchmark strength preservation performance in a dataset of 37 real-world weighted networks^91,92^. The networks span multiple domains, with 28 networks representing social data, 1 network representing informational data, 2 networks representing biological data, 1 network representing economic data and 5 networks representing transportation data. The networks are also diverse in terms of their basic features: network sizes range from 13 to 1,707 nodes and densities range from 0.3% to 78.31% of connections present.

**Fig. 6:**
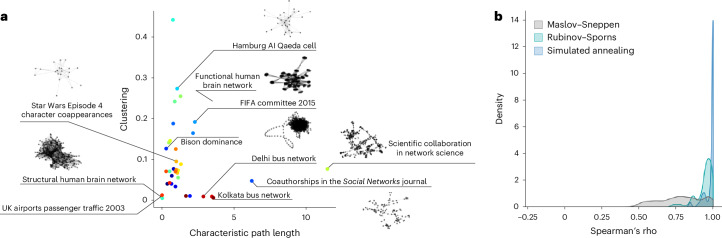
Strength-preserving randomization in real-world networks. **a**, A morphospace spanned by characteristic path length and clustering in which all the weighted real-world networks under study are embedded. A subset of networks are identified and visualized using spring embedding to showcase domain and morphological variability. **b**, A density plot representing Spearman correlation coefficients between strength sequences in empirical and randomized networks derived using the Maslov–Sneppen (gray), Rubinov–Sporns (teal) and simulated annealing algorithms (blue). Source data

Figure 6a shows the networks embedded in a low-dimensional morphospace, illustrating the breadth of network morphologies in the dataset. Figure 6b shows the distributions of Spearman correlation coefficient between strength sequences in empirical and randomized networks. As with brain networks, simulated annealing consistently outperforms both the Rubinov–Sporns (CLES of 83.23%) and Maslov–Sneppen (CLES of 94.82%) algorithms (*P* ≈ 0 for both two-tailed, Wilcoxon–Mann–Whitney two-sample rank-sum tests), with a peak near *ρ* = 1 for most networks in the dataset. This result demonstrates the broad utility of the approach beyond neuroscience.

In Supplementary Information sections ‘Network determinants of simulated annealing performance’ and ‘Influence of strength on global network features’, we further investigate which features of the empirical network impact strength preservation performance in simulated annealing and for which features the choice of a strength-preserving null over their strictly degree-preserving counterparts influences results. Briefly, we find that simulated annealing performance is negatively affected for networks with very low density or heavily right-skewed degree or weight distributions, despite being robust across large portions of the network features considered. Furthermore, by comparing global weighted network features of randomized networks depending on whether only degree or, additionally, strength is preserved, we find that the influence of strength is mostly network-specific. However, in most networks considered, strength-preserving nulls showed higher characteristic path length and clustering, as well as lower assortativity and modularity. This effect was especially strong and consistent for modularity (97% of networks, mean CLES of 93.47%). Therefore, it appears highly prevalent that strength sequence encodes a weaker community structure. Overall, these results show that a network’s strength sequence and, by extension, a network’s weighted nature, can encode information that goes over and above its degree sequence.

## Discussion

Network null models are so ubiquitous in network science that they have been integrated into classic measures such as the small-world coefficient^2,14,15^ and the rich-club coefficient^16,17^. In recent years, graph analysis of brain networks has moved away from earlier binarization procedures to instead consider the large range of edge weights provided by modern imaging and tracing technologies^19,20,23,34,38,39,93^. Weighted graph measures are taking the place of their simpler binary counterparts, allowing researchers to probe the architectural properties characteristic of weighted brain networks^3,9,15^ and the relationships between edge weights and their physical embedding^46,94,95^. Nevertheless, degree-preserving null models remain the most commonly used methods for network statistic normalization^9,18,66,93^. However, such models do not mitigate the possible effects of weighted degree (that is, strength). Efforts toward statistical inference for next-generation connectomics should, therefore, focus on developing and evaluating strength-preserving network null models to adapt to the growing reliance on weighted networks in the field.

Several such null models that preserve a network’s strength sequence, in addition to its degree sequence, have already been developed. One class of strength-preserving models is based on maximum likelihood estimation of exponential random-graph models^45,48–50,96^. They allow unbiased sampling of networks satisfying degree and strength constraints on average but not for each network realization. Furthermore, the generated networks do not preserve the empirical weight distribution^53^. More generally, using a statistical physics nomenclature, the null ensembles resulting from these models can be called ‘canonical’. They embody soft constraints, realized on average over the whole ensemble in contrast to ‘microcanonical’ ensembles, such as the ones approximated in this manuscript, for which hard constraints are satisfied by every network in the ensemble^52,96^. Note that canonical models offer several advantages, including unbiased sampling and computational efficiency^96^. However, they constitute a fundamentally different model, which we consider outside of the scope of this manuscript.

Other alternatives for strength-preserving network randomization include a strength correction procedure that iteratively rescales the weights of a connectivity matrix to obtain any desired strength sequence^46^. An advantage of this method is that it can be easily combined with other constrained network randomization methods to generate surrogate networks embodying multiple constraints. For example, in refs. ^46,79^, the procedure was used in combination with a geometry-preserving network null model. However, this procedure also does not maintain the empirical network’s weight distribution. Finally, in the case of directed networks, the Maslov–Sneppen algorithm has previously been adapted to also preserve the empirical strength sequence^17^ but only of either the incoming or outgoing connections.

Here, we consider a simple strength-preserving randomization procedure that builds upon a rewiring method already widely adopted in the field of network neuroscience^18^. This model is based on weight permutations constrained via simulated annealing^54,55^ and retains the empirical network’s size, density, degree and strength distribution and sequence^72^. We benchmarked this procedure against another strength-preserving randomization algorithm developed by Rubinov and Sporns^65^, as well as the widely used Maslov–Sneppen degree-preserving rewiring. Across multiple performance metrics and data processing choices, simulated annealing stood out as the algorithm that should be favored for strength-preserving randomization.

Moreover, by embedding null network realizations in a two-dimensional morphospace spanned by clustering and characteristic path length, we find that simulated annealing generates less variable morphospace representations than the Rubinov–Sporns algorithm across data processing choices. That is, null networks derived via simulated annealing are more stable in terms of the portion of the morphospace they occupy in relation to nulls generated via other methods, closely aligning with the Maslov–Sneppen null networks. Therefore, once again, the simulated annealing algorithm should probably be preferred to the other two surrogate models for the morphological fidelity of its randomization process and the proximity of the morphological patterns it generates with the well-studied random patterns of the Maslov–Sneppen algorithm.

More broadly, the present report showcases morphospaces as an informative yet underutilized analytical step for understanding the evolution and final position of network architectures as they undergo randomization. Morphospaces have previously been used to relate topological patterns of complex networks in a common space and investigate potential dynamical design principles underlying their architectures^75–79^. Here, we use them to assess null network ensemble variability and feature preservation. By considering null subsamples of increasing size, morphospaces also provide a way to quantify scaling behaviors. An important methodological consideration that emerges from our results is that global weighted network features rapidly converge with the number of generated nulls across all null models, indicating that only a relatively small number of nulls might be necessary for robust normalization of network statistics. Furthermore, using individual participant brain networks, morphospace representation can constitute a tool for the assessment of identity preservation in surrogate networks: by comparing interindividual morphospace distance in empirical and null networks, simulated annealing distinguishes itself as the algorithm that preserves participant identity the best.

We have shown how the simulated annealing algorithm can be applied to preserve degree and strength sequence, but in principle, other features and feature combinations could also be preserved. Using null models that embody a hierarchy of constraints is in fact a fundamental process in network neuroscience^13,46,53,67,94^. When considered in parallel, they allow us to distinguish the additional contribution of higher-order constraints from those inherited from more influential lower-order constraints. For example, geometric constraints can be readily implemented in the simulated annealing objective function, allowing us disentangle the effects of the network’s topology from those passively endowed by spatial embedding^53^.

Similarly, other low-level influential network features such as clustering, motifs or modules could be explored in constraint hierarchies using simulated annealing. Such attempts have been made in the past, in brain networks and various other real-world networks^53,60,61^. However, these models should be thoroughly tested for specific applications, as certain feature combinations might involve optimization tradeoffs. Furthermore, when using rewiring as the basic annealing iteration, certain constraints, such as those imposed on clustering, have been reported to lead to practical breaking of the condition of ergodicity, that is, architectures are formed during rewiring that cannot be destroyed in realistic time frames^52,61^. Null models embodying more complex constraints might, therefore, benefit from other methods from statistical physics such as multicanonical sampling using the Wang–Landau algorithm^52,97,98^.

The present findings should be considered in the context of some methodological limitations. First, while there exists a broad range of edge weight quantification schemes in modern connectomics, we only focused on weight metrics based on streamline counts. However, we find that the performance of simulated annealing generalized to a broad range of real-world complex networks. Therefore, we hypothesize that it should also be the case for other connectome edge weight metrics. Second, while the datasets covered a broad range of possible processing choices, they did not allow to delineate their individual effects on null model performance. As tools for multiverse analysis in connectomics are developed to facilitate the isolation of specific processing choices across a range of pipelines^73^, future work can increasingly interrogate how they affect connectomes and downstream network analysis, including null network generation. As a first step in this direction, we related the strength preservation performance of simulated annealing to various network features in a dataset of diverse real-world networks. We find that strength reconstruction is more difficult for networks with very low density or heavily right-skewed degree or weight distributions, despite being robust across a large portion of the network landscape. Finally, here we use the Maslov–Sneppen degree-preserving rewiring as the randomization step because it is widely used in network science (and particularly in network neuroscience). However, this procedure has been shown to be biased, that is, it does not sample the microcanonical ensemble uniformly in cases of strong degree heterogeneity^52,96,99^. Note, however, that alternatives that uniformly sample the desired ensemble exist^99–106^. Furthermore, the simulated annealing procedure is modular and can easily be used in conjunction with any underlying randomization method.

## Methods

### Data acquisition and network reconstruction

All analyses were applied on two datasets acquired and preprocessed independently. Namely, we used structural connectivity measures derived from data collected at the Lausanne University Hospital^69^ and as part of the HCP S900 release^70^. Together, these data span a variety of methodological choices, allowing us to asses the robustness of the results. These differences in methodology include the use of multiple parcellation resolutions in a structural and a functional atlas for the Lausanne and the HCP datasets, respectively.

#### Lausanne dataset

The Lausanne dataset consists of data collected in *n* = 70 healthy participants (age 28.8 ± 8.9 years old, 37% female) that were scanned at the Lausanne University Hospital in a 3 T Siemens Trio MRI scanner using a 32-channel head coil^69^. The protocol included (1) a magnetization-prepared rapid acquisition gradient echo (MPRAGE) sequence sensitive to white/gray matter contrast (1 mm inplane resolution, 1.2 mm slice thickness) and (2) a DSI sequence (128 diffusion-weighted volumes and a single b0 volume, maximum *b* value of 8,000 s mm^−2^, 2.2 × 2.2 × 3.0 mm^3^ voxel size). Informed written consent was provided by all participants in accordance with institutional guidelines and the protocol was approved by the Ethics Committee of Clinical Research of the Faculty of Biology and Medicine, University of Lausanne, Switzerland.

For each participant, structural connectivity networks were reconstructed using deterministic streamline tractography. The nodes were defined according to a multiscale gray matter parcellation^107^. In the present work, we use a fine 1,000 cortical regions parcellation and a relatively coarser 219 nodes resolution. The FreeSurfer version 5.0.0 open-source package was employed to segment white matter and gray matter from the MPRAGE volumes, whereas tools from the Connectome Mapper open-source software^108^ were used to preprocess DSI data. For each white matter voxel, 32 streamline propagations were initiated per diffusion direction^109^. Structural connectivity was defined as the streamline density between node pairs, that is, the number of streamlines between two regions normalized by the mean length of the streamlines and the mean surface area of the regions^110^. Additional information regarding MRI data preprocessing and network reconstruction is available at ref. ^69^.

Group-representative structural networks were then generated to amplify the signal-to-noise ratio using functions from the netneurotools open-source package (https://netneurotools.readthedocs.io/en/latest/index.html). We adopted a consensus approach that preserves (1) the mean density across participants and (2) the participant-level edge length distribution^71^. First, the cumulative edge length distribution across individual participants’ structural connectivity matrices is divided into *M* bins, with *M* corresponding to the average number of edges across participants. The edge occurring most frequently across participants is then selected within each bin, breaking ties by selecting the edge with the highest average weight. This procedure was performed separately for intra- and interhemispheric edges to ensure that the latter are not under-represented. The selected edges constitute the distance-dependent group-consensus structural network. Finally, the weight of each edge is computed as the mean across participants.

To examine the effect of network density on null model process time, we considered the 219 nodes resolution. We scaled the average number of intra- and interhemispheric edges across participants separately to generate a range of *M* values (that is, 3,602, 3,962, 4,356, 4,794, 5,276, 5,706, 6,162, 6,550, 6,952, 7,346, 7,824, 8,206, 8,596, 9,006, 9,410, 9,862, 10,324, 10,742, 11,180, 11,564, 11,978, 12,460, 12,874, 13,310, 13,622, 14,108 and 14,492). These values were used in the distance-dependent consensus procedure and 100 null networks were generated per *M* value for each null model.

Inversely, to test the effect of network size (number of nodes) on null model process time, we use four increasingly fine parcellations of the Cammoun multiscale atlas^107^, yielding group-consensus networks of 114, 219, 448 and 1,000 cortical nodes. In turn, the number of edges (which effectively drives the effect of density on algorithm process time) is maintained constant with an *M* value of 10,786. Again, the distance-dependent consensus procedure was used for each size and 100 null networks per size were generated for each null model.

For the participant-level analyses, we fixed the density of all individual networks to that of the most sparse network by pruning connections with the lowest weights. This allows for unbiased interindividual comparisons of global network statistics.

#### HCP dataset

MRI data from *n* = 327 unrelated healthy participants (age 28.6 ± 3.73 years old, 55% female) were used to reconstruct structural connectivity networks and build a group-representative connectome^111^. The participants were scanned at Washington University in the HCP’s custom 3-Tesla Siemens ‘Connectome Skyra’ MRI scanner. The protocol included (1) a MPRAGE sequence (repetition time = 2,400 ms, echo time = 2.14 ms, field of view = 224 mm × 224 mm, voxel size = 0.7 mm^3^, 256 slices) and (2) a spin-echo echo-planar imaging sequence (repetition time = 5,520 ms, echo time = 89.5 ms, field of view = 210 mm × 180 mm, voxel size = 1.25 mm^3^, *b*-value of 1,000, 2,000 and 3,000 s mm^−2^, 270 diffusion directions, 18 b0 volumes). Informed written consent was provided by all participants and the protocol was approved by the Washington University Institutional Review Board. Additional information regarding data acquisition is available at ref. ^70^.

The HCP minimal preprocessing pipelines^112^ were applied to the MRI data, and streamline tractography tools from the MRtrix3 open-source software^113^ were used to reconstruct structural connectivity networks in individual participants from diffusion-weighted MRI data. The MPRAGE volume was segmented into white matter, gray matter and cerebrospinal fluid to perform anatomically constrained tractography. Gray matter was divided according to the 400 and 800 cortical regions resolutions of the Schaefer functional parcellation^114^. The multishell, multitissue constrained spherical deconvolution algorithm from MRtrix3 was used to generate fiber orientation distributions^115^. The tractogram was initialized with 40 million streamlines and constrained with a maximum tract length of 250 and a fractional anisotropy cutoff of 0.06. A spherical deconvolution-informed filtering procedure (SIFT2) was then applied following Smith et al.^30^ to estimate streamline-wise cross-section multipliers. Additional information regarding MRI data preprocessing and network reconstruction is available at ref. ^116^.

A group-representative weighted structural connectivity matrix was then generated following the same consensus approach used for the Lausanne dataset. Finally, the weights of the group-consensus structural network were log-transformed and scaled between 0 and 1 to reduce variance in strength.

#### *Drosophila*

The *Drosophila* connectome was reconstructed using 12,995 projection neuron images of the female *Drosophila* brain from the FlyCircuit 1.1 database^35,87^. Imaged neurons were labeled with green fluorescent protein (GFP) using genetic mosaic analysis with a repressible cell marker^117^. GFP-labeled neurons were then delineated from whole-brain three-dimensional images. Individual GFP-labeled neurons were coregistered to a female *Drosophila* template brain using an affine transformation. Each neuronal soma was segmented and its nerve fiber skeleton was automatically traced with an algorithm using the soma’s center as the point of origin. The neurons were then divided into 49 local populations with distinct morphological and functional characteristics, which constituted the nodes of the network. These local populations consisted in 43 local processing units and 6 interconnecting units. A local processing unit was defined as a region with its own population of local interneurons whose fibers are circumscribed to this region. This analysis resulted in a weighted, directed network, with the weight of each edge defined as the number of neuron terminals between two populations. Additional information regarding the *Drosophila* connectome reconstruction is available at ref. ^87^.

#### Mouse

The mouse connectome was reconstructed using publicly available data from 461 tract-tracing experiments conducted in wild-type mice by the Allen Institute for Brain Science^34,118^. Analyses were performed using a whole-brain bilaterally symmetric parcellation of 130 regions designed from the Allen Mouse Brain Atlas^34,119^ and the Allen Developing Mouse Brain Atlas^120^. Each experiment consisted in an anterograde tracer injection into one of the 65 regions of the right hemisphere followed by whole-brain (intrahemispheric and interhemispheric) imaging and mapping of axonal projections. The connection weights were defined as normalized connection densities, proportional to the number of axons projecting from one unit volume of the source region to one unit volume of the target region. Nine regions in each hemisphere were discarded due to the absence of experiments, resulting in a connectome composed of 112 nodes and spurious connections were removed using a probabilistic threshold (*P* < 0.01) based on expert visual assessment^34^. Hemispheric symmetry was assumed, and the available interhemispheric projections were used to reconstruct the whole-brain connectome. As information on the tracer injections’ source and target sites was available, this analysis resulted in a weighted, directed network. Additional information regarding the mouse connectome reconstruction is available at refs. ^34,118^.

#### Rat

The rat connectome was reconstructed using >16,000 rat cortical association macroconnection histological reports from >250 systematically curated references in the primary literature, publicly available in the Brain Architecture Knowledge Management System^89,121^. Considered reports were based on experimental tract tracers transported anterogradely, retrogradely, or in both directions within axons. Analyses were performed using 73 cortical regions derived from the hierarchical Swanson-04 nomenclature^89,122^. One of eight ranked qualitative connection weights was assigned to each connection based on the reports with the most accurate and reliable tracer methodology, optimal injection sites and highest anatomical density of tracer labeling (neuronal soma for source and axon terminals for target). The connection weights were then transformed to approximately logarithmically spaced weights in the range [0, 1]. As information on the tracer injections’ source and target sites was available, this analysis resulted in a weighted, directed network. Additional information regarding the rat connectome reconstruction is available at ref. ^89^.

#### Macaque

The macaque connectome was reconstructed using the macroconnectivity CoCoMac database^123^, collating results from tract-tracing studies in macaque monkeys^90^. Analyses were performed using the combined Walker-von Bonin and Bailey atlas^124^, containing 39 nonoverlapping cortical regions. An anatomical tract was included between two brain regions if (1) it was reported in at least five studies in the database and (2) at least 66% of the reports were positive (that is, detected its presence^7^). The edges were weighted between 1 and 3 based on their average reported strength. As information on the tracer injections’ source and target sites was available, this analysis resulted in a weighted, directed network. Additional information regarding the macaque connectome reconstruction is available at ref. ^90^.

#### Complex weighted networks

To benchmark strength sequence-preserving null network models beyond the use-cases of connectomics, we use a diverse dataset of 37 real-world complex weighted networks. These networks were previously used by Oldham et al.^91^ to compare centrality measures and build on top of another dataset of complex networks compiled by Ghasemian et al.^92^. All networks but one originate from the Index of Complex Networks^125^. The final network is a human structural brain network derived from diffusion-weighted MRI data from the HCP (see ref. ^91^ for more details). Overall, the network sizes range from 13 to 1,707 nodes and their densities range from 0.3% to 78.31% of connections present. The dataset bridges a number of distinct data categories, with 28 networks representing social data, 1 network representing informational data, 2 networks representing biological data, 1 network representing economic data and 5 networks representing transportation data.

### Null models

Two network null models for the strength sequence-preserving randomization of weighted networks were considered as part of the current report. Both algorithms also perfectly preserve the original edge weight distribution. The first model was developed by Rubinov and Sporns for signed functional brain networks^65^ and adapted here to strictly positive structural networks. The second model implements a simulated annealing procedure. To our knowledge, it was introduced in ref. ^72^ as a technique for strength sequence-preserving randomization and further explored in directed connectomes for the preservation of multiple network features in ref. ^53^. Python implementations of both algorithms have been made openly available as part of the netneurotools package (https://netneurotools.readthedocs.io/en/latest/api.html#module-netneurotools.networks).

The two strength sequence-preserving randomization models were benchmarked against the Maslov–Sneppen rewiring algorithm that randomly swaps pairs of edges, systematically disrupting the topology of the empirical network, while preserving network size (that is, number of nodes), density (that is, proportion of expressed edges) and degree sequence (that is, number of edges incident to each node) but not strength sequence (sum of edge weights incident to each node)^18^. This model was implemented using the openly available randmio_und_connected function for undirected (symmetric) networks with positive weights from the Python version of the Brain Connectivity Toolbox (https://github.com/aestrivex/bctpy)^126^. When dealing with directed positive networks, randmio_dir_connected was used instead. This function preserves both in- and out-degree sequences, as well as the out-strength but not the in-strength sequence. These functions have the additional advantage of maintaining connectedness in the rewired network, that is, no node is disconnected from the rest of the network. This constraint is also met in both strength sequence-preserving null models, because they operate on networks rewired according to the Maslov–Sneppen algorithm, simply rearranging weights atop the randomized binary network scaffold to further maintain strength sequence. Finally, for undirected, signed networks (networks containing both positive and negative weights), we used the connection-switching method^127^ to randomize the empirical network while preserving both positive and negative degree sequences. This model was implemented using the openly available randmio_und_signed function from the Python version of the Brain Connectivity Toolbox (https://github.com/aestrivex/bctpy)^126^. For all these models, we specified approximately ten swaps per edge.

The Rubinov–Sporns algorithm^65^ consists of the following steps: First, the randomized network weights are instantiated with zeros ${\hat{A}}_{ij}=0$. Second, the original edge weights are ranked by magnitude. Third, the edges of the randomized network are ranked by the expected weight magnitude ${\hat{e}}_{ij}\propto ({s}_{i}-{\sum }_{u}{\hat{A}}_{iu})({s}_{j}-{\sum }_{u}{\hat{A}}_{ju})$, where *s*_*i*_ is the original strength of node *i*. Fourth, a random edge is selected in the randomized network, and its weight ${\hat{A}}_{ij}$ is set to the original edge weight of the same rank. Finally, the associated edge and weight are removed from further consideration and the remaining original edge weights and randomized network edges are again ranked as described above. The procedure is repeated until every edge in the randomized network has been assigned one of the original weights. The Rubinov–Sporns algorithm was originally developed for signed networks, as implemented in the openly available null_model_und_sign function of the Brain Connectivity Toolbox (https://sites.google.com/site/bctnet)^65^. The original implementation was built on top of the connection-switching method^127^ to randomize the empirical network, while preserving both positive and negative degrees. The weight reassignment procedure was then separately applied to positive and negative edges in the randomized network. Here, for the main analyses applied on structural brain networks with strictly positive weights, Maslov–Sneppen degree-preserving rewiring^18^ is used and the weight reassignment procedure is applied to all edges at once, given that negative weights/strengths did not have to be accounted for.

The last null model employs simulated annealing to preserve the empirical network’s strength sequence. Simulated annealing is a stochastic search algorithm that approximates the global minimum of a given function^54,55^ using the Metropolis technique^128^, controlled by the temperature parameter *T*. A high temperature regime allows the exploration of costly system configurations, whereas fine-tuned adjustments with smaller effects on the system cost are provided at lower temperatures. Initially, the simulated annealing algorithm is set at a high temperature, preventing the process from getting stuck in local minima. Throughout the procedure, the system is slowly cooled down while descending along the optimization landscape, yielding increasingly limited uphill rearrangements. Here, we minimize the cost function *E* defined as the mean squared error between the strength sequence vectors of the original and the randomized networks. When randomizing directed networks, we adapt the cost function by separately computing mean squared error for the in-strengths and the out-strengths and summing both results to obtain *E*. Similarly, when randomizing signed networks, we adapt the cost function by separately computing mean squared error for the positive and the negative strengths and summing both results to obtain *E*. To optimize this function, random weight pairs are swapped. The reconfigurations were only accepted if they lowered the cost of the system or met the probabilistic Metropolis acceptance criterion: $r < \exp (-({E}^{{\prime} }-E)/T\;)$, where *r* ∼ *U*(0, 1). The annealing schedule consisted in 100 stages of 10,000 permutations with an initial temperature of 1,000, halved at each stage. The simulated annealing procedure does not entail any assumption regarding the nature of the edge weights. The signed implementation accepts both positive and negative weights of any kind and will preserve both positive and negative strength sequences. In contrast, the two other implementations accept both positive and negative weights but are naive to their nature, that is, the empirical strength sequence will be preserved but not independently for positive and negative strengths. Pseudocode detailing the complete strength sequence-preserving simulated annealing algorithm is available as Supplementary Algorithm 1.

For directed networks, we also propose an alternative implementation where, instead of swapping a pair of edge weights at random among every possible edge, the basic iteration consists in picking a node at random and swapping a pair of edge weights at random only among its outgoing connections. In this case, the cost function to be minimized is defined as the mean squared error between the in-strength sequence vectors of the original and the randomized networks only. Given that the out-strength sequence is already preserved by the Maslov–Sneppen algorithm and that we only permute outgoing edge weights node-wise, the out-strength sequence remains preserved and the algorithm instead focuses on reconstructing the in-strength sequence of the original network.

For the participant-level analysis, performance is evaluated at each stage and the annealing schedule is halted if performance reaches a predefined energy threshold of 0.0001 or runs for a maximum of 1,000 stages. This threshold was chosen to ensure a uniform final energy distribution across nulls of different empirical networks and avoid interindividual differences in downstream analyses due to differences in the quality of the null strength reconstruction.

Importantly, when generating the null network ensembles, we first generate the Maslov–Sneppen rewired networks. The strength-preserving procedures are then applied to each rewired network individually and the optimal solution is retained. Individual null networks, therefore, share the same topology across all the algorithms considered. Only their weight assignments differ.

### Performance metrics

To evaluate the strength sequence reconstruction performance of the network null models under study, the Spearman rank-order correlation coefficient was mainly used, whereas the Kolmogorov–Smirnov statistic was used as a measure of the distance between the empirical and null strength distributions. To assess the differences between measures derived from distinct network null models, we used the nonparametric Mann–Whitney *U* rank test. To assess the effect size, we used the CLES, an intuitive measure that directly relates to the Mann–Whitney *U* (ref. ^129^). CLES corresponds to the proportion of all possible pairs between two groups that support a given direction, for example, correlation coefficients between empirical and null strength sequences are larger using the simulated annealing algorithm than the Rubinov–Sporns algorithm. It can be interpreted as the probability that a randomly selected value from one ensemble is greater than a randomly selected value from the other ensemble. The Spearman correlation coefficient, Kolmogorov–Smirnov statistic and Mann–Whitney *U* rank test were computed using the corresponding functions from the scipy open-source package (https://docs.scipy.org/doc/scipy/index.html)^130^, whereas CLES was computed using the compute_effsize function from the pingouin open-source package (https://pingouin-stats.org/build/html/index.html)^131^.

Below we detail the other performance metrics used in this study, namely, the simulated annealing objective function (mean squared error) and computational cost (process time) operationalizations:

- Mean squared error. In the context of this study, the mean squared error (m.s.e.) is defined as
  1 $${\mathrm{m.s.e.}}=\frac{1}{n}\mathop{\sum }\limits_{i = 1}^{n}{({s}_{i}-{\hat{s}}_{i})}^{2},$$
  where *n* is the number of nodes in the network, *s*_*i*_ is the strength of node *i* and ${\hat{s}}_{i}$ is the strength of node *i* in the randomized network.
- Process time. The process times reported in this study strictly correspond to central processing unit execution times of the randomization processes in seconds. They were computed on a machine equipped with 2.20 GHz Intel Xeon Gold 5320 processors. Specifically, process times were computed as the differences between consecutive calls to the process_time function of the Python Standard Library time module. The calls to the process_time function were placed immediately before and after the calls to the randomization algorithms. While the algorithms were mostly run in parallel using the joblib open-source package (https://joblib.readthedocs.io/en/stable/) to reduce their walltimes, the algorithms were run in single processes when evaluating the process times reported in this manuscript to ensure accuracy. To relate process time to the density or size of the network to be randomized, the process time of the Maslov–Sneppen rewiring was subtracted from the process time of the two strength-preserving randomization algorithms, because they both incorporate the procedure, which depends on the number of edges and nodes in the network.

### Topological features

- Degree. Degree corresponds to the number of edges incident on a node.
- Strength. Weighted degree or strength corresponds to the sum of edge weights incident on a node.
- Clustering coefficient. The weighted local clustering coefficient of a node corresponds to the mean ‘intensity’ of triangles around a node. The clustering coefficient *C* of a node *u* can be defined as^132^
  2 $${C}_{u}=\frac{2}{{k}_{u}({k}_{u}-1)}\sum _{ij}{({w}_{ui}{w}_{ij}{w}_{ju})}^{\frac{1}{3}},$$
  where *k*_*u*_ is the degree of node *u* and *w*_*ij*_ is the weight of the edge incident on nodes *i* and *j*, scaled by the largest weight in the network. It was computed using the openly available clustering_coef_wu function from the Python version of the Brain Connectivity Toolbox (https://github.com/aestrivex/bctpy)^126^. Before applying the function, the weights of the network were normalized to the range [0, 1] using the weight_conversion function from the Python version of the Brain Connectivity Toolbox (https://github.com/aestrivex/bctpy)^126^. The network average clustering coefficient was computed as the mean across weighted local clustering coefficients of all nodes in the network.
- Characteristic path length. In all networks considered, a larger weight indicates greater importance. Therefore, to compute weighted shortest paths, we first define a monotonic transformation from edge weight to edge length, which can be intuitively considered as the cost of traversing an edge. We use the negative natural logarithm to map edge weights to lengths in all human connectomes and an inverse transformation for all other networks to account for various weight ranges. Dijkstra’s algorithm^133^ was then used to identify the sequence of unique edges spanning the minimum length between each node pair (that is, the shortest path). Shortest path lengths were then computed as the sums of traversed edges’ lengths. This procedure was implemented using the openly available distance_wei function from the Python version of the Brain Connectivity Toolbox (https://github.com/aestrivex/bctpy)^126^. The characteristic path length of a network was finally computed as the average across all shortest path lengths in the network.
- Assortativity coefficient. The assortativity coefficient *r* of a network corresponds to the Pearson correlation coefficient between the strengths of connected nodes^134^. Using the openly available assortativity_wei function from the Python version of the Brain Connectivity Toolbox (https://github.com/aestrivex/bctpy)^126^, it is evaluated as
  3 $$r=\frac{{M}^{-1}{\sum }_{i}{s}_{i}{t}_{i}-{\left[{M}^{-1}{\sum }_{i}\frac{1}{2}({s}_{i}+{t}_{i})\right]}^{2}}{{M}^{-1}{\sum }_{i}\frac{1}{2}\left({s}_{i}^{2}+{t}_{i}^{2}\right)-{\left[{M}^{-1}{\sum }_{i}\frac{1}{2}({s}_{i}+{t}_{i})\right]}^{2}},$$
  where *s*_*i*_ and *t*_*i*_ are the strengths of the nodes at the ends of the *i*th edge, with *i* = 1, …, *M.*
- Modularity. To compute network modularity *Q*, we used the Louvain modularity maximization algorithm^135^ to detect nonoverlapping communities of nodes that maximize the weight of within-community edges and minimize the weight of between-community edges. The quality function used was
  4 $$Q=\frac{1}{2m}\sum _{ij}\left({w}_{ij}-\frac{{s}_{i}{s}_{j}}{2m}\right)\delta ({c}_{i},{c}_{j}),$$
  where *m* is the total weight of all edges in the network, *w*_*ij*_ is the weight of the edge incident on nodes *i* and *j*, *s*_*i*_ is the strength of node *i*, *c*_*i*_ is the community assignment of node *i* and *δ*(*c*_*i*_, *c*_*j*_) is the Kronecker delta function and is equal to 1 when *c*_*i*_ = *c*_*j*_ and 0 otherwise. The algorithm was applied to the network 250 times using the consensus_modularity function from the netneurotools open-source package (https://netneurotools.readthedocs.io/en/latest/index.html), and its final modularity was computed as the average of the resulting optimized modularities *Q*.

### Hub identification

Network hubs are a set of centrally embedded nodes^136^. When considering (weighted) degree as the centrality measure in question, the presence of hubs in a network is often detected on the basis of the presence of a heavy-tail degree distribution. In contrast, an exponentially decaying degree distribution is consistent with expectations from random networks^137^.

Parametric modeling of degree distributions is often used to detect heavy tailedness. However, these methods depend on subjective processing choices. Therefore, following Gajwani et al.^73^, we opt for a nonparametric approach to facilitate comparison across datasets and null models^74^.

Briefly, we assess the heavy tailedness of a weighted degree distribution by determining how heavy its right tail is in contrast to what is expected of the exponential distribution, that is, whether it shows subexponential tail decay. To do so, we compute the empirical first and third quartiles, denoted as *Q*1 and *Q*3, respectively, along with the interquartile range (IQR; IQR = *Q*3 − *Q*1). The measure of ‘right tailedness’ is then defined as the probability that a randomly selected observation from the distribution exceeds the value represented by *Q*3 + 3 × IQR (that is, *p*_*R*_(*X*) = *P*(*X* > *Q*3 + 3 × IQR)), a widely used definition of outliers^138^. This computed probability can be contrasted with the right tailedness of the exponential distribution (*X* ≈ e^−*λx*^), which remains constant across different shape parameters (*λ*). Specifically, *p*_*R*_(*X*) ≈ 0.009 (ref. ^74^), and heavy tailedness is detected if the empirical *p*_*R*_ exceeds this analytic threshold.

Hubs are defined as the outliers (values exceeding *Q*3 + 3 × IQR), and the *z*-scored Rand index is used^139^ to measure the similarity of the partitions of network nodes into hubs and nonhubs between empirical networks and their randomized nulls.

### Weighted rich-club detection

A rich club is defined as a set of high-degree nodes (rich nodes) that share more connections than expected by chance^16,80^. In this study, to further account for the relative strength of rich links, that is, links connecting rich nodes, we consider a weighted measure of the rich-club phenomenon, the weighted rich-club coefficient^17,81^, which takes the general form

5 $${\phi }^{w}(k)=\frac{C}{D},$$

where *C* = *W*_≥*k*_ is the weighted connectedness of the club, that is, the total weight of the rich links, and *D* is a denominator which captures the maximal possible weighted connectedness of the club under a specific null hypothesis. The rich nodes are defined as nodes with degree ≥*k*, and a range of *k* values are considered.

Considering that high-degree nodes have a higher chance of being interconnected, the (weighted) rich-club coefficient is typically normalized against a null rich-club coefficient computed in a population of randomized null networks^9,17,81^. While the classic Maslov–Sneppen degree-preserving rewiring^9,10,18^ is often used to build the null network population even when computing the weighted rich-club coefficient, it does not account for the effect of weighted degree sequence on the weighted rich-club phenomena.

To contrast inferences obtained using different null models, we compute the weighted rich-club coefficient in each of the 10,000 null networks generated for each null model under study. We then compute the normalized weighted rich-club coefficient as

6 $${\phi }_{\mathrm{norm}}^{w}(k)=\frac{{\phi }^{w}(k)}{{\phi }_{\mathrm{rand}}^{w}(k)},$$

with ${\phi }_{\mathrm{rand}}^{w}(k)$ the mean weighted rich-club coefficient across the null network population.

Following^81^, equation (6) can also be simplified as

7 $${\phi }_{\mathrm{norm}}^{w}(k)=\frac{C}{{C}_{\mathrm{rand}}},$$

with *C* = *W*_≥*k*_ the weighted connectedness of the empirical club and *C*_rand_ the mean weighted connectedness across the null network population. As reported in ref. ^81^, the denominator *F* in equation (5) can take multiple forms. Given a network null model that preserves the empirical richness (degree) sequence and weight distribution, many of these definitions lead to the empirical and null denominators canceling out when computing the normalized weighted rich-club coefficient. Equation (6) can then be simplified into equation (7), effectively only accounting for the numerator in equation (5). For more details, see ref. ^81^.

To test the robustness of our results, we also consider a specific denominator *F* for equation (5), which now takes the form

8 $${\phi }^{w}(k)=\frac{{W}_{\ge k}}{\mathop{\sum }\nolimits_{i = 1}^{{E}_{\ge k}}{w}_{i}^{\mathrm{rank}}},$$

where *E*_≥*k*_ is the number of rich links, and ${w}_{i}^{\mathrm{rank}}$ is the weight of the network edge at rank *i*, given a ranking by weight. We chose this definition because of its wide use in the network science literature, including in seminal papers on the rich-club phenomenon in the human connectome^9,10,17^. This version of the weighted rich-club coefficient measures the proportion of weight that rich nodes share in relation to the overall amount they could potentially share if linked by the network’s most robust connections. We use it to compute normalized rich-club coefficients across a subsample of 100 null networks.

Finally, to assess the statistical significance of the weighted rich-club phenomenon, a *P* value is derived as the proportion of null *ϕ*^*w*^(*k*) that are greater than the empirical *ϕ*^*w*^(*k*).

### Reporting summary

Further information on research design is available in the Nature Portfolio Reporting Summary linked to this article.

## Supplementary information

Supplementary Information Supplementary results, Algorithm 1 and Figs. 1–21.

Reporting Summary

## Source data

Source Data Fig. 2 Brillouin light scattering spectra at three current values.

Source Data Fig. 3 Phase relation (experimental data and the fitting curves).

Source Data Fig. 4 Evolution of relative phase (experiments and simulation).

Source Data Fig. 6 Evolution of relative phase versus gate voltage (simulation).

## Data Availability

Original and intermediate, preprocessed, data used in this work are available at https://github.com/netneurolab/milisav_strength_nulls (ref. ^140^). The Lausanne dataset is available at Zenodo via https://zenodo.org/records/2872624 (ref. ^69^). The HCP dataset^70^ is available at https://db.humanconnectome.org/data/projects/HCP_1200. The *Drosophila* connectome is available at 10.1016/j.cub.2015.03.021 (ref. ^87^). The Allen Mouse Connectivity^34,118^ (connectivity.brain-map.org), Developing Mouse Brain^34,119^ (developingmouse.brain-map.org) and Mouse Brain^120^ (mouse.brain-map.org) atlases are made openly available by the Allen Institute for Brain Science. The CoCoMac database^123^ is available at http://cocomac.g-node.org. The rat connectome^89^ is available at https://bams1.org/connectomes/standard_rat.php. The dataset of real-world complex weighted networks^91,92,125^ is available at https://figshare.com/s/22c5b72b574351d03edf?file=25762442. Source data for Figs. 2b, 3, 4 and 6 are available with this paper. Large Source data files for Figs. 2a and 5 are available at 10.5281/zenodo.13988937 (ref. ^141^).

## Code availability

The Python code used to perform the analyses and generate the figures presented in this paper is available at https://github.com/netneurolab/milisav_strength_nulls (ref. ^140^).
