# Supplementary material for: A simulated annealing algorithm for randomizing weighted networks
Source: Nat Comput Sci. 2024 Dec 10;5(1):48–64. doi: 10.1038/s43588-024-00735-z (PMC11774763; doi:10.1038/s43588-024-00735-z)
Supplement: Supplementary file 1 — Supplementary results, Algorithm 1 and Figs. 1–21. [file 43588_2024_735_MOESM1_ESM.pdf]

nature computational science

Supplementary information https://doi.org/10.1038/s43588-024-00735-z

A simulated annealing algorithm for randomizing weighted networks

In the format provided by the authors and unedited

Table-of-contents

SUPPLEMENTARY INFORMATION . . . **2**
Null model calibration . . . 2
Alternative objective function . . . 2
Alternative annealing schedule . . . 2
Log-transformation . . . 3
Computational cost . . . 3
Morphospace trajectories . . . 4
Weighted rich-club inference and geometry . . . 4
Alternative strength-preserving randomization for directed networks . . . 4
Strength-preserving randomization for signed networks . . . 5
Network determinants of simulated annealing performance . . . 5
Influence of strength on global network features . . . 6

References . . . **7**

SUPPLEMENTARY INFORMATION

Null model calibration

Scatter plots of strengths in the empirical and the randomized networks provide additional information about individual null model calibration and bias, i.e., the fidelity of its behavior across the range of data under consideration. For example, in the HCP dataset (Fig. 2a, S7), the Rubinov–Sporns algorithm appears to systematically underestimate low strengths and overestimating high strengths, as shown by the preponderance of data points below the identity line for lower empirical strength values and above the identity line for higher empirical strength values. By comparison, the Maslov–Sneppen algorithm shows a more even spread of points around the identity line, and even slightly outperforms the Rubinov–Sporns algorithm in terms of mean squared error (MSE; Fig. S1) in the low resolution HCP network ($p < 10^{-22}$, CLES $= 54.08\%$, two-tailed, Wilcoxon–Mann–Whitney two-sample rank-sum test). Importantly, the simulated annealing algorithm yields data points which perfectly align to the identity line, indicative of an unbiased fit. In contrast, in the Lausanne dataset (Fig. S7), the Maslov–Sneppen model underestimates high strengths, with most data points falling under the identity line at high empirical strength values. Conversely, the Rubinov–Sporns algorithm and the simulated annealing algorithm exhibit a number of data points above the identity line. In the case of simulated annealing, a small number of low strengths are over-estimated. Importantly, these errors do not appear to be systematic, as they affect different regions in different null realizations. Therefore, screening strength preservation results for each null realization could avoid introducing a bias towards the overestimation of low strengths in the null ensemble without incurring a considerably longer processing time. Furthermore, these low-strength outliers only have a small effect on simulated annealing performance, as shown by the high correlation coefficients obtained.

Alternative objective function

To mitigate the small reconstruction errors observed in the Lausanne dataset using simulated annealing, we consider a different objective function that penalizes large individual errors: maximum absolute error. We explore the effect of this objective function in 100 null networks for each empirical network. We find that low-strength outliers are less apparent but that this effect is achieved at the detriment of the overall fit of the model (Fig. S2). For all networks, the strength sequence preservation is considerably worse as assessed using Spearman rank-order correlation (LAU - low res: $p < 10^{-33}$, CLES $= 85.10\%$, LAU - high res: $p < 10^{-65}$, CLES $= 100\%$, HCP - low res: $p < 10^{-68}$, CLES $= 100\%$, HCP - high res: $p < 10^{-66}$, CLES $= 100\%$, two-tailed, Wilcoxon–Mann–Whitney two-sample rank-sum test).

Alternative annealing schedule

The annealing schedule is the process used to control the temperature during simulated annealing [2, 20]. In the *Results* section, we only present analyses in which a geometric cooling schedule was used for the simulated annealing algorithm (see *Methods* for more details). While this is the most widely used annealing schedule [2, 29], alternatives exist, including non-monotonic schedules. Here, we explore the use of a non-monotonic annealing schedule, which allows reheating when the system gets stuck in a local minimum [2, 31, 32].

While monotonic cooling strategies allow the system to escape from local minima (see Fig. 1c and *Methods* for more details), a situation may arise where the system, under simulated annealing, converges to an inadequate super-structure and gets stuck in a local minimum under a low temperature regime. The costly rearrangements required to exit this local minimum and allow super-structural changes would then be highly improbable. The system could then be reheated to escape the local minimum and allow super-structural changes. Here, we explore this approach by modifying our simulated annealing algorithm to detect local minima and reheat the system. Specifically, a local minimum was detected if no weight swap was accepted in an annealing stage and the temperature was updated according to: $T_{i+1} = \max(T_r/2, T_b)$, where T_{i+1} is the temperature at iteration $i + 1$, T_r is the previous reheat temperature, and T_b is the temperature at which the best solution to date was obtained. When no local minimum is detected, temperature is decreased according to the standard geometric cooling schedule used in the main analyses: $T_{i+1} = \alpha T_i$.

To test this new annealing schedule, we use the low-resolution Lausanne connectome. Applying the reheating strategy with the default annealing parameters used in the main analyses (100 stages of 10000 iterations, with $\alpha = 0.5$) resulted in the same null networks. This is due to the fact that no local minimum was detected, i.e., all annealing stages resulted in at least one weight swap. In Fig. S4a, we show the energy of the optimal solution (blue) as a function of the annealing stage (throughout the simulated annealing procedure) for an example null network. We observe a similar trajectory as in Fig. S13c (see section "Morphospace trajectories" for more details). However, to show that local minima can be escaped even without reheating, we overlay the energy trajectory of the optimal solution with the actual energy of the system at the end of each annealing stage (yellow). We see that considerable increases in energy can occur, but that they are largely constrained to the early stages of the procedure, as explained above.

Next, since no strict local minimum (absence of swap

in a stage) was detected under the default parameters, we shorten the annealing stages (1000 iterations per stage instead of 10000) to induce local minima and reheating. In order for the results to be comparable (to maintain the same total number of iterations), we concomitantly increase the number of stages to 1000. Finally, so that temperature does not decrease too fast given the increased number of stages, we use $\alpha = 0.9$. With these updated parameters, we find that two local minima are detected throughout the procedure, leading to reheating (Fig. S4b, bottom) and apparent escapes (concomitant high increases in energy, as seen in Fig. S4b, top). We then generate two ensembles of 100 null networks: one using the default annealing schedule and the other using reheating. We compare the resulting energies (MSE) of the optimal solutions and find that the reheating schedule produces a better reconstruction of the empirical strength sequence ($p = 0.004, CLES = 61.85\%$, two-tailed, Wilcoxon–Mann–Whitney two-sample rank-sum test). To ensure that this effect is not simply due to slower cooling, we compare the energies of the reheated ensemble to that of an ensemble that underwent the same updated annealing schedule (1000 stages of 1000 iterations with $\alpha = 0.9$), but without reheating. Again, we find that the reheating schedule outperforms the monotonic cooling schedule ($p = 5.23 \times 10^{-5}, CLES = 66.56\%$, two-tailed, Wilcoxon–Mann–Whitney two-sample rank-sum test).

In summary, using a non-monotonic annealing schedule can yield a better reconstruction of the empirical strength sequence, but might require more parameter fine-tuning.

Log-transformation

Our assessment of the quality of strength reconstruction revealed a slight tendency of the simulated annealing algorithm to overestimate low strengths in networks with a heavily right-skewed weight distribution. This could be due to a combination of high weight outliers and low-strength nodes having lower degrees and therefore providing fewer “degrees of freedom” in matching their original strength through weight permutations. We posit that the small discrepancies in strength reconstruction observed between datasets might be due to a simple but potentially influential processing step: log-transformation, which was applied to the HCP dataset but not the Lausanne dataset. This common practice consists in taking the logarithm of the network’s edge weights and scaling them between 0 and 1 [6, 34]. Weight distributions of physical connections between neural elements have often been described as approximately log-normal across scales and species [5, 8, 26]. Log-transformation is therefore believed to bring a network’s weight distribution closer to normality, making it more amenable to downstream analyses. In the case of the simulated annealing algorithm, correcting the strong skewness of connectome weight distributions might allow for more degrees of freedom in reconstructing the strength sequence, consequently leading to faster convergence. In line with this hypothesis, we find that applying the log-transformation to the Lausanne dataset leads to better solutions (LAU - low res: $p < 10^{-26}$, CLES $= 93.31\%$, LAU - high res: $p < 10^{-33}$, CLES $= 100\%$) of similar quality to those obtained with the HCP dataset (Fig. S3).

Computational cost

The results so far show that simulated annealing outperforms alternative network null models at reconstructing the empirical strength sequence. Given the pervasive assumption that simulated annealing is time consuming, we next sought to benchmark the computational cost associated with each procedure. Unlike the other two algorithms, simulated annealing naturally involves a tradeoff between computational cost and performance. Namely, the user specifies the number of iterations per annealing stage—more iterations result in better solutions but this comes at the cost of added execution time. To examplify this, we use the low-resolution Lausanne dataset for computational efficiency. Fig. S5 (left) shows that MSE is logarithmically reduced with the number of iterations per annealing stage (blue), with a concomitant linear increase in execution time (yellow). To illustrate the benefit of added iterations, Fig. S5 (right) shows strength sequence fits at a small (1000) and large ($100\,000$) number of iterations. We see that low-strength inaccuracies which are present for a small number of iterations get “ironed off” as we increase the number of iterations. Importantly, simulated annealing’s flexible nature allows it to reach arbitrarily optimal solutions with a sufficiently slow cooling schedule [1, 2, 4, 7, 10, 12, 13, 17, 28, 36, 47]. However, while on average, each null took approximately 3 seconds to generate using 1000 iterations, it took approximately 98 seconds to generate using $100\,000$ iterations. Generating a complete null ensemble could therefore require several hours without parallelization. Nevertheless, simulated annealing’s strength reconstruction performance initially improves rapidly with an increase in iterations, providing adequate reconstruction at the default value of $10\,000$ iterations (see Fig. S7, top).

Given that simulated annealing is more computationally intensive, how feasibly can it be applied to more fine-grained networks with a greater number of edges? Would users be forced to use the Maslov–Sneppen or Rubinov–Sporns algorithms in those instances? Fig. S6a (left) shows execution time for the three null model algorithms run on empirical networks with increasing density. Note that the duration of the Maslov–Sneppen rewiring was subtracted from that of the Rubinov–Sporns and simulated annealing procedures because the two strength-preserving weight reassignment procedures are

modular. Here, they build atop the Maslov–Sneppen algorithm, but they could effectively be used in combination with any randomization procedure. Considering their execution time separately allows us to focus on their specific scaling behaviors while maintaining an equal footing. First, we observe that the Maslov–Sneppen and Rubinov–Sporns algorithms have considerably shorter execution time. However, we find that simulated annealing scales well, showing a near-flat relation with increasing density. By comparison, the Rubinov–Sporns algorithm shows a slightly worse scaling behavior, characterized by a steeper slope. Finally, the execution time of Maslov–Sneppen rewiring is the most sensitive to network density. Importantly, the Maslov–Sneppen algorithm's strength reconstruction performance concomitantly decreases, as shown by an increase in MSE as a function of network density (Fig. S6a, right). In contrast, the Rubinov–Sporns and simulated annealing algorithms' performances are not affected by density. Similar results were observed when considering the relationships between network size (number of nodes) and the algorithms' execution time and performance (Fig. S6b). Overall, these results show the flexibility of the simulated annealing procedure: despite greater computational cost, simulated annealing can be tuned to achieve even better performance, and it scales well to more detailed networks.

Morphospace trajectories

A question that might arise when using iterative optimization methods such as simulated annealing is how dependent the solution is on the number of iterations. Once again, morphospace representation can be used to address this question. Namely, it allows us to ask how much do global architectural features of null networks change as a function of optimization performance or number of steps taken. As an example, we consider the low-resolution empirical networks of the Lausanne and HCP dataset and we track the trajectory of a null network through morphospace during optimization (Fig. S13a,d). We find that while early optimization steps result in big leaps in the morphospace, i.e. highly variable solutions depending on the moment the process is stopped, displacements quickly localize to a constrained portion of the morphospace. Correspondingly, we observe a rapid transition in performance relatively early in the simulated annealing process (Fig. S13c,f). This indicates that simulated annealing reaches a high level of performance early on and that the following minor increases in performance do not result in important structural changes for the null networks. In other words, each null network, based on the simulated annealing procedure's random initialization, has a predetermined place in the morphospace. This is further confirmed by aggregating morphospace positions and performance (in terms of MSE) across 100 null trajectories of 100 annealing stages (Fig. S13b,e). Indeed, we find a clear dichotomy between an area of low-energy and an area of high-energy. Therefore, tracking performance and variability in the null network features under consideration for a subset of nulls could be very useful in establishing an iteration threshold or performance target to ultimately reduce the duration of the null networks' generation, while still preserving a good approximation.

Weighted rich-club inference and geometry

In section "The weighted rich-club phenomenon", we have previously shown that using simulated annealing-derived null networks yields larger normalized rich-club ratios than using Rubinov–Sporns or Maslov–Sneppen randomization (Fig. 4, left, S14, top; $p < 0.01$ for all two-tailed, Wilcoxon–Mann–Whitney two-sample rank-sum tests). We posited that the differences in normalized rich-club coefficient observed between models was due to an overestimation of strength in high-degree nodes. Here, we verify this hypothesis and further relate this phenomenon to the prevalence of long-distance connections between hubs [44] and the exponential decay of connection weights with physical distance in structural brain networks [35, 42]. First, we consider the difference between the normalized rich-club coefficient obtained using simulated annealing and that obtained using Maslov–Sneppen rewiring. We relate this measure to median average weight (ratio of strength to degree) across the rich nodes. We find a strong negative relationship between the two measures (LAU - low res: $\rho = -0.96, p < 10^{-27}$, LAU - high res: $\rho = -0.70, p < 10^{-11}$, HCP - low res: $\rho = -0.99, p < 10^{-47}$, HCP - high res: $\rho = -0.96, p < 10^{-41}$). This indicates that the difference in normalized rich-club coefficient between the two models might indeed be due to an overestimation of strength in high-degree nodes by the Maslov–Sneppen rewiring (Fig. S14; middle). We then relate median average weight to the median Euclidean distance across rich connections. Again, we find strong negative relationships between the two (LAU - low res: $\rho = -0.91, p < 10^{-18}$, LAU - high res: $\rho = -0.93, p < 10^{-32}$, HCP - low res: $\rho = -0.87, p < 10^{-19}$, HCP - high res: $\rho = -0.62, p < 10^{-8}$), indicative of the previous effect possibly being due to a preponderance of low-weight long-range connections between rich nodes (Fig. S14; bottom). Similar results were obtained for an alternative definition of the weighted rich-club coefficient [30, 45] (see *Methods* and Fig. S15 for more details).

Alternative strength-preserving randomization for directed networks

In this implementation, instead of swapping a pair of edge weights at random among every possible edge, the basic iteration consists in picking a node at random and

swapping a pair of edge weights at random only among its outgoing connections. Furthermore, the cost function to be minimized is now defined as the mean squared error between the in-strength sequence vectors of the empirical and the randomized networks only. Given that the out-strength sequence is already preserved by the Maslov–Sneppen algorithm and that we only permute outgoing edge weights node-wise, the out-strength sequence remains preserved and the algorithm instead focuses on reconstructing the in-strength sequence of the empirical network. We find that this alternative implementation also provides better results than the previous one in terms of in-strength sequence reconstruction for all animal connectomes except macaque (drosophila: $p \approx 0$, CLES = 72.31%; mouse: $p \approx 0$, CLES = 79.06%; rat: $p \approx 0$, CLES = 77.33%; macaque: $p < 10^{-11}$, CLES = 51.41%, two-tailed, Wilcoxon–Mann–Whitney two-sample rank-sum test, see Fig. S17 for scatter plots of strengths in the empirical and the randomized networks). This is potentially due to the reduced search space being easier to explore despite providing less "degrees of freedom".

Strength-preserving randomization for signed networks

Many real-world networks are signed (include both positive and negative weights). Here we adapt strength-preserving simulated annealing to signed networks by iteratively applying the algorithm to the positive and negative edges separately. This allows us to maintain both the empirical positive and negative strength sequences, as well as the positive and negative weight distributions. Instead of applying the procedure atop Maslov–Sneppen degree-preserving nulls, we use the connection-switching method [50] to randomize the empirical network while preserving both positive and negative degrees. This method was implemented using the openly available *randmio_und_signed* function from the Python version of the Brain Connectivity Toolbox (https://github.com/aestrivex/bctpy) [37], specifying approximately 10 swaps per edge.

To assess algorithm performance, we apply strength-preserving simulated annealing to a dataset of signed brain networks reflecting inter-regional similarity across seven local biological features (https://github.com/netneurolab/hansen_many_networks) [15]. These networks include: correlated gene expression (reconstructed using the microarray Allen Human Brain Atlas [16, 24]), neurotransmitter receptor similarity (reconstructed using positron emission tomography [14, 25]), laminar similarity (reconstructed using the Merker-stained BigBrain neuron density atlas [3, 33]), metabolic connectivity (reconstructed using dynamic FDG-PET [18, 19, 49]), haemodynamic connectivity (reconstructed using resting-state functional magnetic resonance imaging - fMRI [46]), electrophysiological connectivity (reconstructed using resting-state magnetoencephalography [22, 23, 40, 46]), and temporal profile similarity (a measure of dynamic similarity reconstructed from fMRI time series features [9, 41]). Nodes were defined based on the Schaefer functional parcellation [39] at a resolution of 400 cortical regions (see [15], for details on acquisition and preprocessing of each dataset). Additionally, we benchmark the performance of the simulated annealing algorithm against that of the connection-switching method [50] and the original Rubinov–Sporns algorithm for signed networks, using the openly available *null_model_und_sign* function of the Brain Connectivity Toolbox (https://sites.google.com/site/bctnet) [37].

For each of the 7 inter-regional similarity networks (Fig. S19, top row), we generate 100 null networks according to each algorithm. To assess the performance of the null models in preserving strength sequence, we plot empirical network strengths against strengths of the randomized networks for all 100 nulls. Fig. S19 shows the resulting scatterplots for positive strengths (top row) and negative strengths (bottom row) separately for each algorithm (grey: connection-switching, teal: Rubinov–Sporns, blue: simulated annealing). We also compute Spearman rank-order correlation coefficients between the empirical strength sequence and the strength sequence of each null. We find that across all the considered brain networks, simulated annealing shows the best performance ($p \approx 0$, CLES $= 100\%$ for all networks, two-tailed, Wilcoxon–Mann–Whitney two-sample rank-sum test), with a near-perfect reconstruction of the empirical strength sequence ($M \approx 1.0$ for all networks). In contrast, the connection-switching method showed highly variable and biased performance across networks, with mean absolute correlation coefficients ranging from 0.056 to 0.987.

In conclusion, we show that the simulated annealing algorithm can be readily adapted to signed networks to preserve both positive and negative strength sequences. Note however that the use of correlation as a connectivity measure has been associated with the emergence of non-random topological features, such as increased clustering. These features are not necessarily captured by network null models that are naive to the underlying connectivity measure, such as the ones presented in the current report. Network null models should therefore be used cautiously when dealing with correlation networks and results should be interpreted in the context of the null hypothesis the nulls embody. For a detailed overview of pitfalls and solutions associated with the use of null models for correlation networks, see [51].

Network determinants of simulated annealing performance

While most networks show a near-perfect reconstruction of the empirical strength sequence using simulated annealing, Fig. 6b shows a small number of outliers

with slightly lower performance. We have previously observed low-strength inaccuracies using the Lausanne dataset and some directed animal connectomes. We hypothesized these errors to arise from the heavily right-skewed weight distributions of these networks and from low-strength nodes having lower degrees and therefore providing fewer "degrees of freedom" in reconstructing the strength sequence (see section "Strength-preserving randomization for directed networks" and Supplementary Information section "Log-transformation" for more details). Here we test these hypotheses across a wide variety of real-world networks to determine how network features affect the simulated annealing algorithm's performance. Specifically, in Fig. S20a, we plot the performance of each of the 37 real-world networks under consideration (mean Spearman's rho for strength reconstruction across 100 null networks) against various network features. These features include the normality and skewness of the weight distribution (in line with the previous observation that normally distributed weights led to better performance than right-skewed weights), the uniformity and skewness of the degree distribution, as well as the network density (in line with the "degrees of freedom" hypothesis). Normality is assessed using the Shapiro–Wilk statistic, whereas uniformity is assessed using the Kolmogorov–Smirnov statistic, following a generalized Monte Carlo goodness-of-fit procedure, implemented using the *goodness_of_fit* function from the scipy open-source package (https://docs.scipy.org/doc/scipy/index.html) [11, 21, 43, 48]. We define outliers as the networks for which performance was lower than the 20th percentile of the performance distribution and plot their empirical strength sequences against the strength sequences of their randomized counterparts (Fig. S20b).

We find that across most network features, strength reconstruction performance is robust across a large portion of the feature ranges, with performance outliers mostly concentrated at the extremes of these feature ranges. This indicates that simulated annealing is robust across a wide-range of network features but that its performance becomes more variable at extremes. Moreover, the absence of clear relationships across all network features indicates that simulated annealing performance might instead be explained by a confluence of multiple features. In general, we find that networks with a weight distribution closer to normality show a better performance, that performance drops for networks with very low density and that outliers generally show higher degree heterogeneity and right-skewed degree and weight distributions (Fig. S20a). Furthermore, scatterplots of strength sequences in empirical and randomized outlier networks generally show a higher propensity for low-strength inaccuracies (Fig. S20b). Overall, these results align with previous observations and the notion that network features which lower the algorithm's "degrees of freedom" lower performance.

Influence of strength on global network features

In the previous section, we have investigated the network determinants of simulated annealing performance. Inversely, we can test how the simulated annealing algorithm influences network features. In other words, when and how much does strength matter? Specifically, we compare global network features of randomized networks depending on whether only degree or, additionally, strength is preserved. We consider characteristic path length (CPL), mean clustering, assortativity, and modularity and quantify the difference between the distributions of degree and strength-preserving nulls using the common-language effect size: the probability that a randomly selected value from the degree-preserving ensemble is greater than a randomly selected value from the strength-preserving ensemble (Fig. S21). We find that the direction of the effect varies as a function of the network considered. This indicates that the constraints that strength exerts on global network features vary across network classes. Nevertheless, we find that characteristic path length and mean clustering mostly show higher values in the strength-preserving nulls (66% of networks for CPL, mean CLES = 35.73% and 86% of networks for mean clustering, mean CLES = 24.96%). This indicates that across multiple network classes, weight assignment that matches the empirical strength sequence results in less efficient communication pathways but increased weighted clustering. In contrast, changes in assortativity were smaller between the two null models and no clear direction of the effect emerged across networks (60% of networks showed higher values for degree-preserving nulls, mean CLES = 58.11%), suggesting that the influence of strength on assortativity is more network-specific than for the other global features considered. Finally, we observe a strong and highly consistent effect towards higher modularity in degree-preserving nulls as compared with strength-preserving nulls across diverse network classes (97% of networks, mean CLES = 93.47%). Therefore it appears highly prevalent that strength sequence encodes a weaker community structure. Overall, these results show that a network's strength sequence, and by extension, a network's weighted nature, can encode information that goes over and above its degree sequence. Therefore, strength-preserving null models can yield different inferences than strictly degree-preserving null models and importantly, when used in combination, such a hierarchy of constraints can disentangle the specific contribution of strength to network organization.

[1] Aarts, E. and Korst, J. (1989). *Simulated annealing and Boltzmann machines: a stochastic approach to combinatorial optimization and neural computing*. John Wiley & Sons, Inc.

[2] Abramson, D., Krishnamoorthy, M., Dang, H., et al. (1999). Simulated annealing cooling schedules for the school timetabling problem. *Asia Pacific Journal of Operational Research*, 16:1–22.

[3] Amunts, K., Lepage, C., Borgeat, L., Mohlberg, H., Dickscheid, T., Rousseau, M.-É., Bludau, S., Bazin, P.-L., Lewis, L. B., Oros-Peusquens, A.-M., et al. (2013). BigBrain: An ultrahigh-resolution 3D human brain model. *Science*, 340(6139):1472–1475.

[4] Anily, S. and Federgruen, A. (1987). Simulated annealing methods with general acceptance probabilities. *Journal of Applied Probability*, 24(3):657–667.

[5] Avena-Koenigsberger, A., Mišić, B., Hawkins, R. X., Griffa, A., Hagmann, P., Goñi, J., and Sporns, O. (2017). Path ensembles and a tradeoff between communication efficiency and resilience in the human connectome. *Brain Structure and Function*, 222(1):603–618.

[6] Bazinet, V., Hansen, J. Y., Vos de Wael, R., Bernhardt, B. C., van den Heuvel, M. P., and Misic, B. (2023). Assortative mixing in micro-architecturally annotated brain connectomes. *Nature Communications*, 14(1):2850.

[7] Bertsimas, D. and Tsitsiklis, J. (1993). Simulated annealing. *Statistical Science*, 8(1):10–15.

[8] Buzsáki, G. and Mizuseki, K. (2014). The log-dynamic brain: How skewed distributions affect network operations. *Nature Reviews Neuroscience*, 15(4):264–278.

[9] Fulcher, B. D. and Jones, N. S. (2017). hctsa: A computational framework for automated time-series phenotyping using massive feature extraction. *Cell Systems*, 5(5):527–531.

[10] Geman, S. and Geman, D. (1984). Stochastic relaxation, Gibbs distributions, and the Bayesian restoration of images. *IEEE Transactions on Pattern Analysis and Machine Intelligence*, (6):721–741.

[11] Genest, C. and Rémillard, B. (2008). Validity of the parametric bootstrap for goodness-of-fit testing in semiparametric models. In *Annales de l'IHP Probabilités et statistiques*, volume 44, pages 1096–1127.

[12] Gidas, B. (1985). Nonstationary Markov chains and convergence of the annealing algorithm. *Journal of Statistical Physics*, 39:73–131.

[13] Hajek, B. (1988). Cooling schedules for optimal annealing. *Mathematics of Operations Research*, 13(2):311–329.

[14] Hansen, J. Y., Shafiei, G., Markello, R. D., Smart, K., Cox, S. M., Nørgaard, M., Beliveau, V., Wu, Y., Gallezot, J.-D., Aumont, É., et al. (2022). Mapping neurotransmitter systems to the structural and functional organization of the human neocortex. *Nature Neuroscience*, pages 1–13.

[15] Hansen, J. Y., Shafiei, G., Voigt, K., Liang, E. X., Cox, S. M., Leyton, M., Jamadar, S. D., and Misic, B. (2023). Integrating multimodal and multiscale connectivity blueprints of the human cerebral cortex in health and disease. *PLOS Biology*, 21(9):e3002314.

[16] Hawrylycz, M. J., Lein, E. S., Guillozet-Bongaarts, A. L., Shen, E. H., Ng, L., Miller, J. A., Van De Lagemaat, L. N., Smith, K. A., Ebbert, A., Riley, Z. L., et al. (2012). An anatomically comprehensive atlas of the adult human brain transcriptome. *Nature*, 489(7416):391–399.

[17] Henderson, D., Jacobson, S. H., and Johnson, A. W. (2003). The theory and practice of simulated annealing. *Handbook of metaheuristics*, pages 287–319.

[18] Jamadar, S. D., Ward, P. G., Close, T. G., Fornito, A., Premaratne, M., O'Brien, K., Stäb, D., Chen, Z., Shah, N. J., and Egan, G. F. (2020). Simultaneous BOLD-fMRI and constant infusion FDG-PET data of the resting human brain. *Scientific Data*, 7(1):363.

[19] Jamadar, S. D., Ward, P. G., Liang, E. X., Orchard, E. R., Chen, Z., and Egan, G. F. (2021). Metabolic and hemodynamic resting-state connectivity of the human brain: A high-temporal resolution simultaneous BOLD-fMRI and FDG-fPET multimodality study. *Cerebral Cortex*, 31(6):2855–2867.

[20] Kirkpatrick, S., Gelatt Jr, C. D., and Vecchi, M. P. (1983). Optimization by simulated annealing. *Science*, 220(4598):671–680.

[21] Kojadinovic, I. and Yan, J. (2012). Goodness-of-fit testing based on a weighted bootstrap: A fast large-sample alternative to the parametric bootstrap. *Canadian Journal of Statistics*, 40(3):480–500.

[22] Liu, Z.-Q., Luppi, A. I., Hansen, J. Y., Tian, Y. E., Zalesky, A., Yeo, B. T., Fulcher, B. D., and Misic, B. (2024). Benchmarking methods for mapping functional connectivity in the brain. *bioRxiv*, pages 2024–05.

[23] Liu, Z.-Q., Shafiei, G., Baillet, S., and Misic, B. (2023). Spatially heterogeneous structure-function coupling in haemodynamic and electromagnetic brain networks. *NeuroImage*, 278:120276.

[24] Markello, R. D., Arnatkeviciute, A., Poline, J.-B., Fulcher, B. D., Fornito, A., and Misic, B. (2021). Standardizing workflows in imaging transcriptomics with the abagen toolbox. *eLife*, 10:e72129.

[25] Markello, R. D., Hansen, J. Y., Liu, Z.-Q., Bazinet, V., Shafiei, G., Suárez, L. E., Blostein, N., Seidlitz, J., Baillet, S., Satterthwaite, T. D., et al. (2022). Neuromaps: Structural and functional interpretation of brain maps. *Nature Methods*, 19(11):1472–1479.

[26] Markov, N. T., Ercsey-Ravasz, M. M., Ribeiro Gomes, A., Lamy, C., Magrou, L., Vezoli, J., Misery, P., Falchier, A., Quilodran, R., Gariel, M.-A., et al. (2014). A weighted and directed interareal connectivity matrix for macaque cerebral cortex. *Cerebral Cortex*, 24(1):17–36.

[27] Maslov, S. and Sneppen, K. (2002). Specificity and stability in topology of protein networks. *Science*, 296(5569):910–913.

[28] Mitra, D., Romeo, F., and Sangiovanni-Vincentelli, A. (1986). Convergence and finite-time behavior of simulated annealing. *Advances in Applied Probability*, 18(3):747–771.

[29] Nourani, Y. and Andresen, B. (1998). A comparison of simulated annealing cooling strategies. *Journal of Physics A: Mathematical and General*, 31(41):8373.

[30] Opsahl, T., Colizza, V., Panzarasa, P., and Ramasco, J. J. (2008). Prominence and control: The weighted rich-club effect. *Physical Review Letters*, 101(16):168702.

[31] Osman, I. H. (1993). Metastrategy simulated annealing and tabu search algorithms for the vehicle routing problem. *Annals of Operations Research*, 41:421–451.

[32] Osman, I. H. (1995). Heuristics for the generalised as-

signment problem: simulated annealing and tabu search approaches. *Operations-Research-Spektrum*, 17:211–225.

[33] Paquola, C., Vos De Wael, R., Wagstyl, K., Bethlehem, R. A., Hong, S.-J., Seidlitz, J., Bullmore, E. T., Evans, A. C., Misic, B., Margulies, D. S., et al. (2019). Microstructural and functional gradients are increasingly dissociated in transmodal cortices. *PLOS Biology*, 17(5):e3000284.

[34] Park, B.-y., de Wael, R. V., Paquola, C., Larivière, S., Benkarim, O., Royer, J., Tavakol, S., Cruces, R. R., Li, Q., Valk, S. L., et al. (2021). Signal diffusion along connectome gradients and inter-hub routing differentially contribute to dynamic human brain function. *NeuroImage*, 224:117429.

[35] Roberts, J. A., Perry, A., Lord, A. R., Roberts, G., Mitchell, P. B., Smith, R. E., Calamante, F., and Breakspear, M. (2016). The contribution of geometry to the human connectome. *NeuroImage*, 124:379–393.

[36] Romeo, F. and Sangiovanni-Vincentelli, A. (1991). A theoretical framework for simulated annealing. *Algorithmica*, 6:302–345.

[37] Rubinov, M. and Sporns, O. (2010). Complex network measures of brain connectivity: Uses and interpretations. *NeuroImage*, 52(3):1059–1069.

[38] Rubinov, M. and Sporns, O. (2011). Weight-conserving characterization of complex functional brain networks. *NeuroImage*, 56(4):2068–2079.

[39] Schaefer, A., Kong, R., Gordon, E. M., Laumann, T. O., Zuo, X.-N., Holmes, A. J., Eickhoff, S. B., and Yeo, B. T. (2018). Local-global parcellation of the human cerebral cortex from intrinsic functional connectivity MRI. *Cerebral Cortex*, 28(9):3095–3114.

[40] Shafiei, G., Baillet, S., and Misic, B. (2022). Human electromagnetic and haemodynamic networks systematically converge in unimodal cortex and diverge in transmodal cortex. *PLOS Biology*, 20(8):e3001735.

[41] Shafiei, G., Markello, R. D., De Wael, R. V., Bernhardt, B. C., Fulcher, B. D., and Misic, B. (2020). Topographic gradients of intrinsic dynamics across neocortex. *eLife*, 9:e62116.

[42] Stiso, J. and Bassett, D. S. (2018). Spatial embedding imposes constraints on neuronal network architectures. *Trends in Cognitive Sciences*, 22(12):1127–1142.

[43] Stute, W., Manteiga, W. G., and Quindimil, M. P. (1993). Bootstrap based goodness-of-fit-tests. *Metrika*, 40:243–256.

[44] Van Den Heuvel, M. P., Kahn, R. S., Goñi, J., and Sporns, O. (2012). High-cost, high-capacity backbone for global brain communication. *Proceedings of the National Academy of Sciences*, 109(28):11372–11377.

[45] Van Den Heuvel, M. P. and Sporns, O. (2011). Rich-club organization of the human connectome. *Journal of Neuroscience*, 31(44):15775–15786.

[46] Van Essen, D. C., Smith, S. M., Barch, D. M., Behrens, T. E., Yacoub, E., Ugurbil, K., Consortium, W.-M. H., et al. (2013). The WU-Minn human connectome project: An overview. *NeuroImage*, 80:62–79.

[47] Van Laarhoven, P. J., Aarts, E. H., van Laarhoven, P. J., and Aarts, E. H. (1987). *Simulated annealing*. Springer.

[48] Virtanen, P., Gommers, R., Oliphant, T. E., Haberland, M., Reddy, T., Cournapeau, D., Burovski, E., Peterson, P., Weckesser, W., Bright, J., et al. (2020). SciPy 1.0: Fundamental algorithms for scientific computing in python. *Nature Methods*, 17(3):261–272.

[49] Voigt, K., Liang, E. X., Misic, B., Ward, P. G., Egan, G. F., and Jamadar, S. D. (2023). Metabolic and functional connectivity provide unique and complementary insights into cognition-connectome relationships. *Cerebral Cortex*, 33(4):1476–1488.

[50] Wormald, N. C. et al. (1999). Models of random regular graphs. *London Mathematical Society Lecture Note Series*, pages 239–298.

[51] Zalesky, A., Fornito, A., and Bullmore, E. (2012). On the use of correlation as a measure of network connectivity. *NeuroImage*, 60(4):2096–2106.

Supplementary Algorithm 1 Strength sequence-preserving network randomization

procedure StrPrsvnRand($A, nstage, niter, temp, frac$)
    Compute strengths S from adjacency matrix A
    Compute m, the number of edges in A
    Compute B, a Maslov–Sneppen rewiring of A
    Compute strengths S_b from B
    Compute energy E as a function of S and S_b
    $E_{min} \leftarrow E$
    $B_{curr} \leftarrow B$
    for $stage = 1, 2, \ldots, nstage$ **do**
        for $iteration = 1, 2, \ldots, niter$ **do**
            $e1 \leftarrow RandomInt(1, m)$
            $e2 \leftarrow RandomInt(1, m)$
            Compute B' by permuting $e1$ and $e2$ in B_{curr}
            Compute strengths S'_b from B'
            Compute energy E' as a function of S and S'_b
            $\Delta E \leftarrow E' - E$
            if $\Delta E < 0 \vee Random(0, 1) < exp(\frac{-\Delta E}{temp})$ **then**
                $B_{curr} \leftarrow B'$
                $S_b \leftarrow S'_b$
                $E \leftarrow E'$
                if $E < E_{min}$ **then**
                    $B \leftarrow B_{curr}$
                    $E_{min} \leftarrow E$
                end if
            end if
        end for
        $temp \leftarrow temp \times frac$
    end for
    return B, E_{min}
end procedure

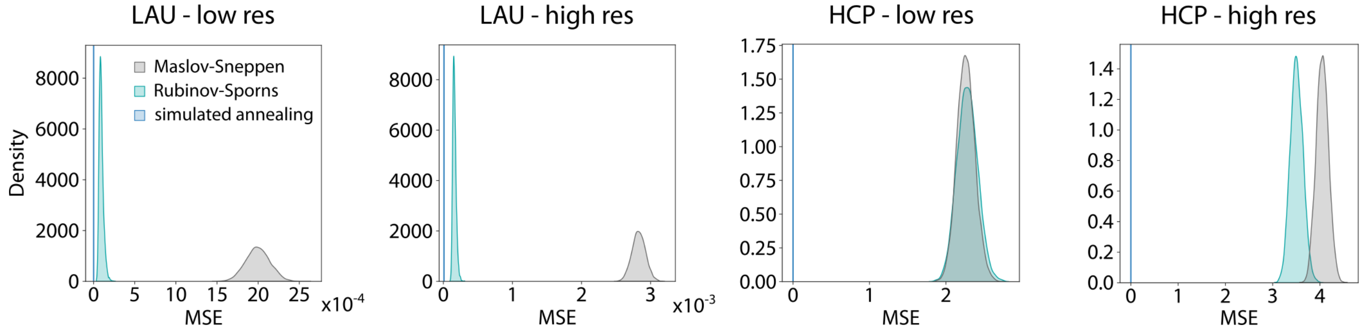

Supplementary Figure S1 **Benchmarking strength sequence preservation - MSE** | Density plots representing mean squared error between the strength sequences of the empirical and the randomized networks for the Maslov–Sneppen algorithm (grey), the Rubinov–Sporns algorithm (teal) and the simulated annealing algorithm (blue). Due to low variability, we only show the mean MSE across null realizations for the simulated annealing algorithm.

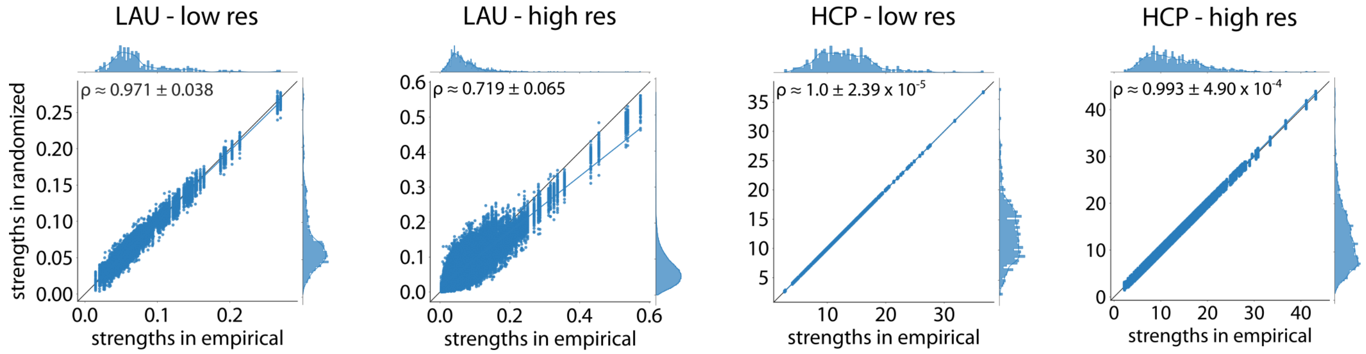

Supplementary Figure S2 **Benchmarking strength sequence preservation - maximum absolute error** | Scatter plots of strengths of the empirical (abscissa) and 100 null networks (ordinate) obtained using simulated annealing with the maximum absolute error objective function. Each point represents a brain region. Marginal distribution histograms are shown on the top and right axes. Mean and standard deviation across 100 Spearman rank-order correlation coefficients are provided as insets. Linear regression lines (blue) are computed over the whole ensemble for visualization purposes. The identity line (black) is provided as reference.

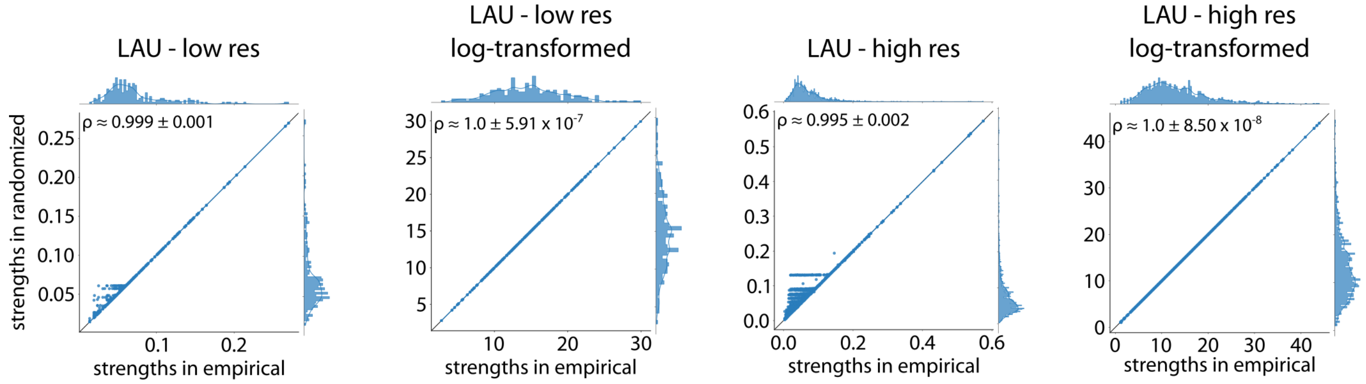

Supplementary Figure S3 **Benchmarking strength sequence preservation - log-transformation** | Scatter plots of strengths of the empirical (abscissa) and 100 null networks (ordinate) for the empirical and the log-transformed Lausanne networks. Each point represents a brain region. Marginal distribution histograms are shown on the top and right axes. Mean and standard deviation across 100 Spearman rank-order correlation coefficients are provided as insets. Linear regression lines (blue) are computed over the whole ensemble for visualization purposes. The identity line (black) is provided as reference.

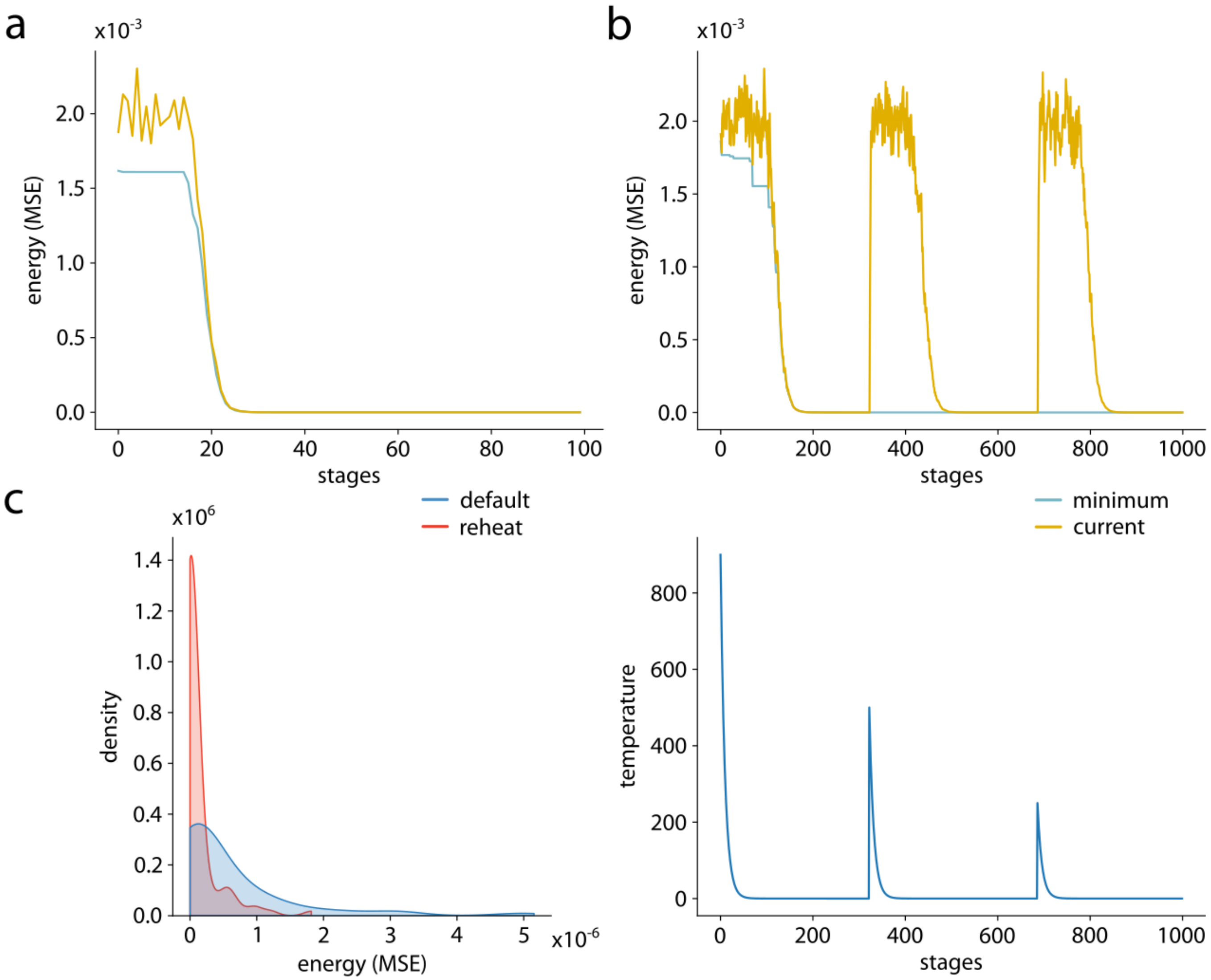

Supplementary Figure S4 **Alternative annealing schedule** | (a) Optimization trajectory (energy as a function of annealing stage) for an example null network using the default monotonic cooling schedule. The energy of the optimal solution (minimum energy) is shown in blue and the energy of the current solution at the end of each stage is shown in yellow. (b) Top: Same as in (a) but for the non-monotonic annealing schedule. Bottom: Corresponding temperature trajectory. (c) Density plot representing energies in terms of MSE across 100 nulls for the monotonic and non-monotonic schedules.

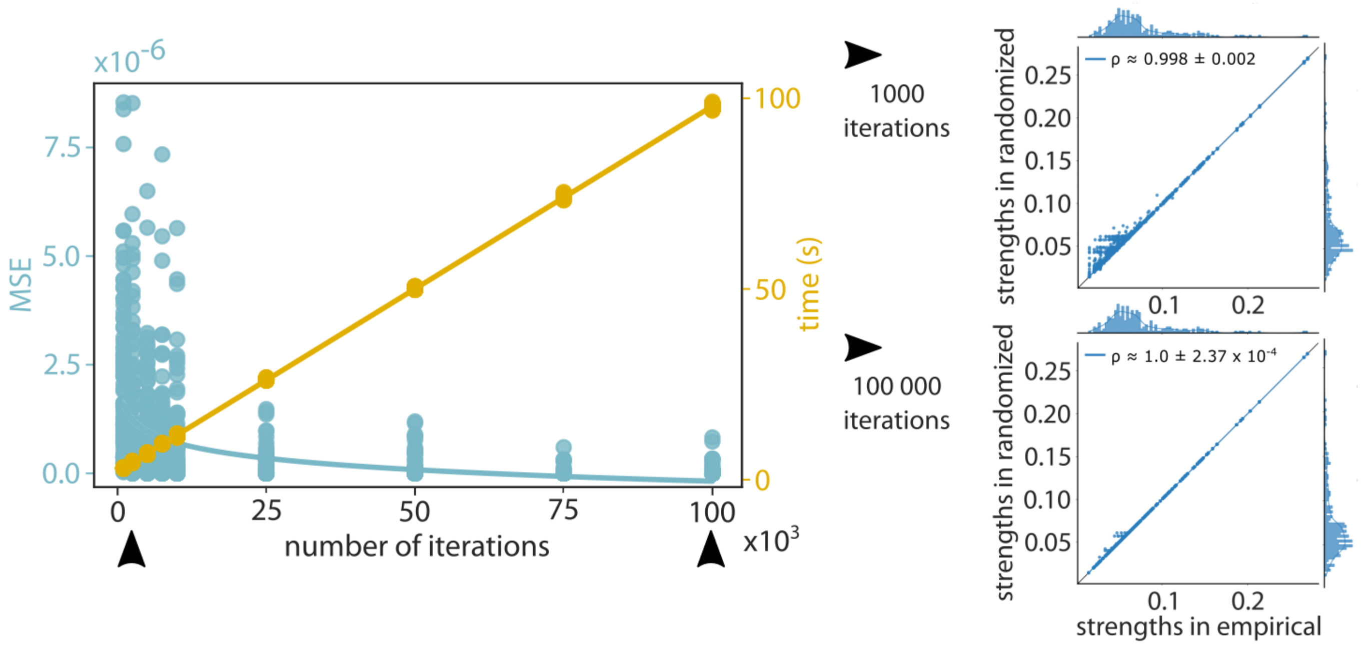

Supplementary Figure S5 **Computational cost-performance tradeoff of the simulated annealing procedure** All analyses were carried out on the low resolution group-representative connectome of the Lausanne dataset. Left: MSE (e) decays logarithmically as a function of the number of iterations per annealing stage (n) while process time (t) increases linearly. The blue line corresponds to the least squares-fitted function $e = -3.82 \times 10^{-7} log(n) + 4.22 \times 10^{-6}, R^2 = 0.22$. The yellow line corresponds to the least squares-fitted function $t = 9.6 \times 10^{-4} n + 2.12, R^2 \approx 1.0$ Right: Scatter plots of strengths of the empirical (abscissa) and randomized (ordinate) networks for all 100 null networks generated via annealing stages implementing 1000 (top) and 100 000 iterations (bottom). Mean and standard deviation across 100 Spearman rank-order correlation coefficients are provided as insets. Linear regression lines (blue) are computed over the whole ensemble for visualization purposes. The identity line (black) is provided as reference. Interestingly, when the algorithm is allowed to run for more iterations, even the minor inaccuracies observed in the Lausanne dataset for low strength nodes (previously shown in Fig. S7) are completely "ironed out", with a near-perfect reconstruction of the empirical strength sequence.

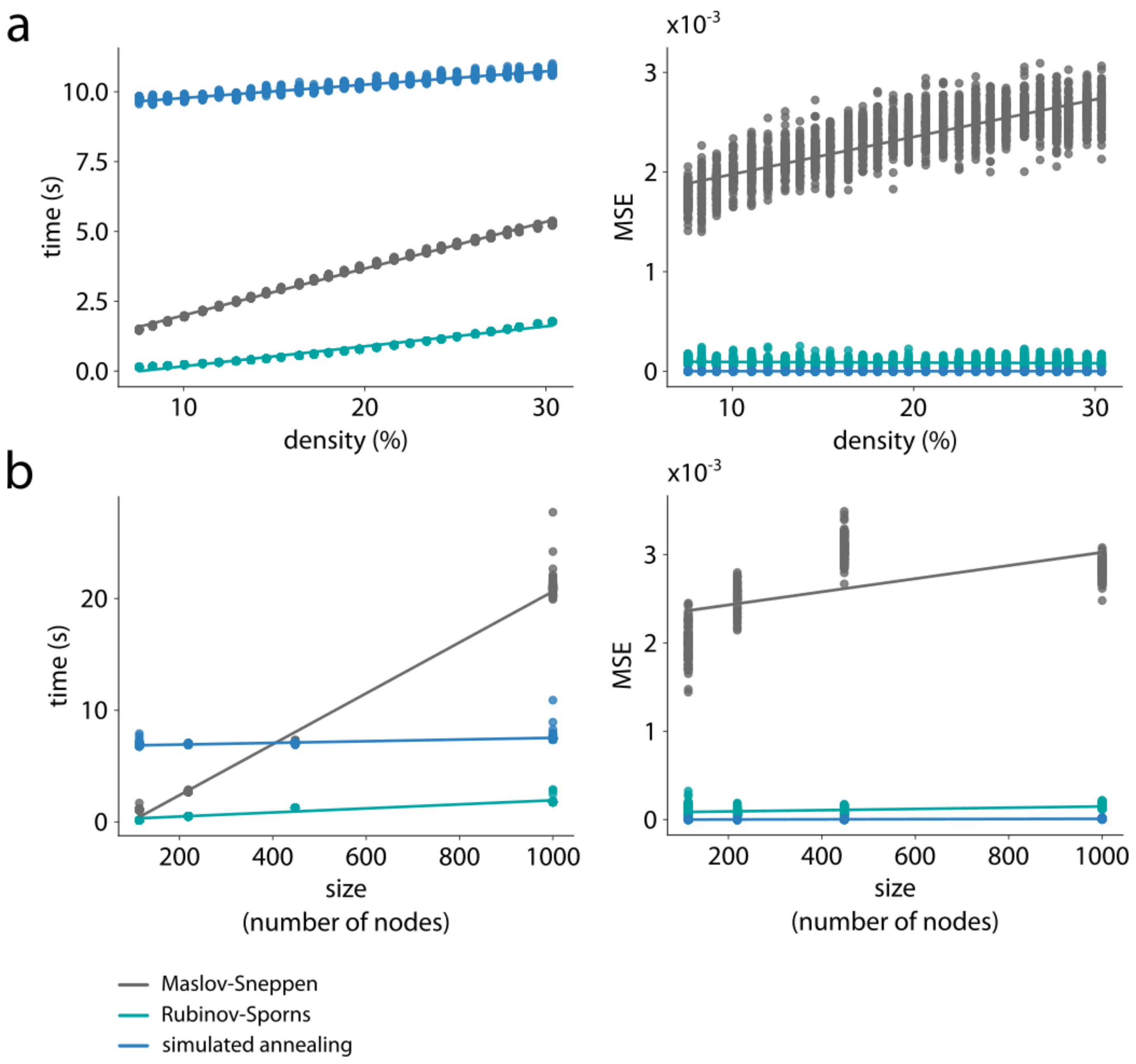

Supplementary Figure S6 **Scaling of the simulated annealing procedure with network density and size** All analyses were carried out on the low resolution group-representative connectome of the Lausanne dataset. Note that the duration of the Maslov–Sneppen rewiring was subtracted from that of the Rubinov–Sporns and simulated annealing procedures. (a) Left: Linear relationships between process time (t) in seconds and density (d) for all three randomization algorithms. The grey line corresponds to $t = 0.1674d + 0.32, R^2 \approx 1.0$. The teal line corresponds to $t = 0.0715d - 0.54, R^2 = 0.98$. The blue line corresponds to $t = 0.0477d + 9.3, R^2 = 0.95$. Right: Linear relationships between performance in terms of MSE (p) and density (d) for all three randomization algorithms. The grey line corresponds to $p = 3.76 \times 10^{-5}d + 0.0016, R^2 = 0.69$. The teal line corresponds to $p = -6.12 \times 10^{-7}d + 9.99 \times 10^{-5}, R^2 = 0.02$. The blue line corresponds to $p = 4.23 \times 10^{-10}d + 6.31 \times 10^{-7}, R^2 = 6.4 \times 10^{-6}$. (b) Left: Linear relationships between process time (t) in seconds and network size (s) for all three randomization algorithms. The grey line corresponds to $t = 0.0228s - 2.16, R^2 = 0.99$. The teal line corresponds to $t = 0.0018s + 0.12, R^2 = 0.9$. The blue line corresponds to $t = 7.5 \times 10^{-4}s + 6.78, R^2 = 0.57$. Right: Linear relationships between performance in terms of MSE (p) and network size (s) for all three randomization algorithms. The grey line corresponds to $p = 7.43 \times 10^{-7}s + 0.0023, R^2 = 0.35$. The teal line corresponds to $p = 7.01 \times 10^{-8}s + 7.87 \times 10^{-5}, R^2 = 0.27$. The blue line corresponds to $p = 9.31 \times 10^{-9}s - 6.44 \times 10^{-7}, R^2 = 0.53$. All functions were fitted using the least squares method. Lines are colored by the null algorithm used (Maslov–Sneppen in grey, Rubinov–Sporns in teal, and simulated annealing in blue).

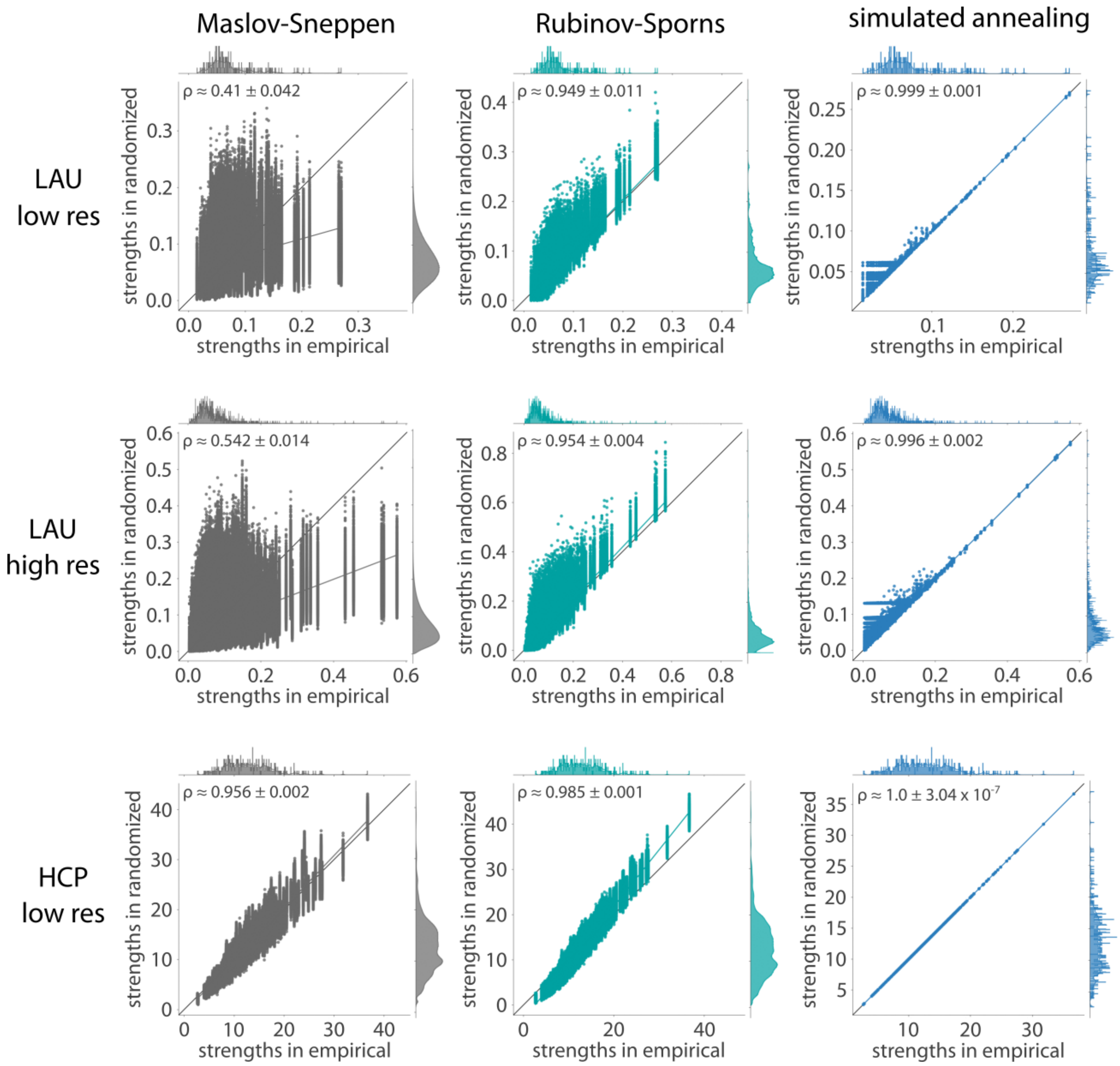

Supplementary Figure S7 **Benchmarking strength sequence preservation** | Scatter plots of strengths of the empirical (abscissa) and randomized (ordinate) networks for all 10 000 null networks, where each point represents a brain region. Marginal distribution histograms are shown on the top and right axes. Mean and standard deviation across 10 000 Spearman rank-order correlation coefficients are provided as insets. Data points and histograms appear in grey for the Maslov–Sneppen algorithm, teal for the Rubinov–Sporns algorithm, and blue for the simulated annealing algorithm. Linear regression lines (colored) are computed over the whole ensemble for visualization purposes. The identity line (black) is provided as reference.

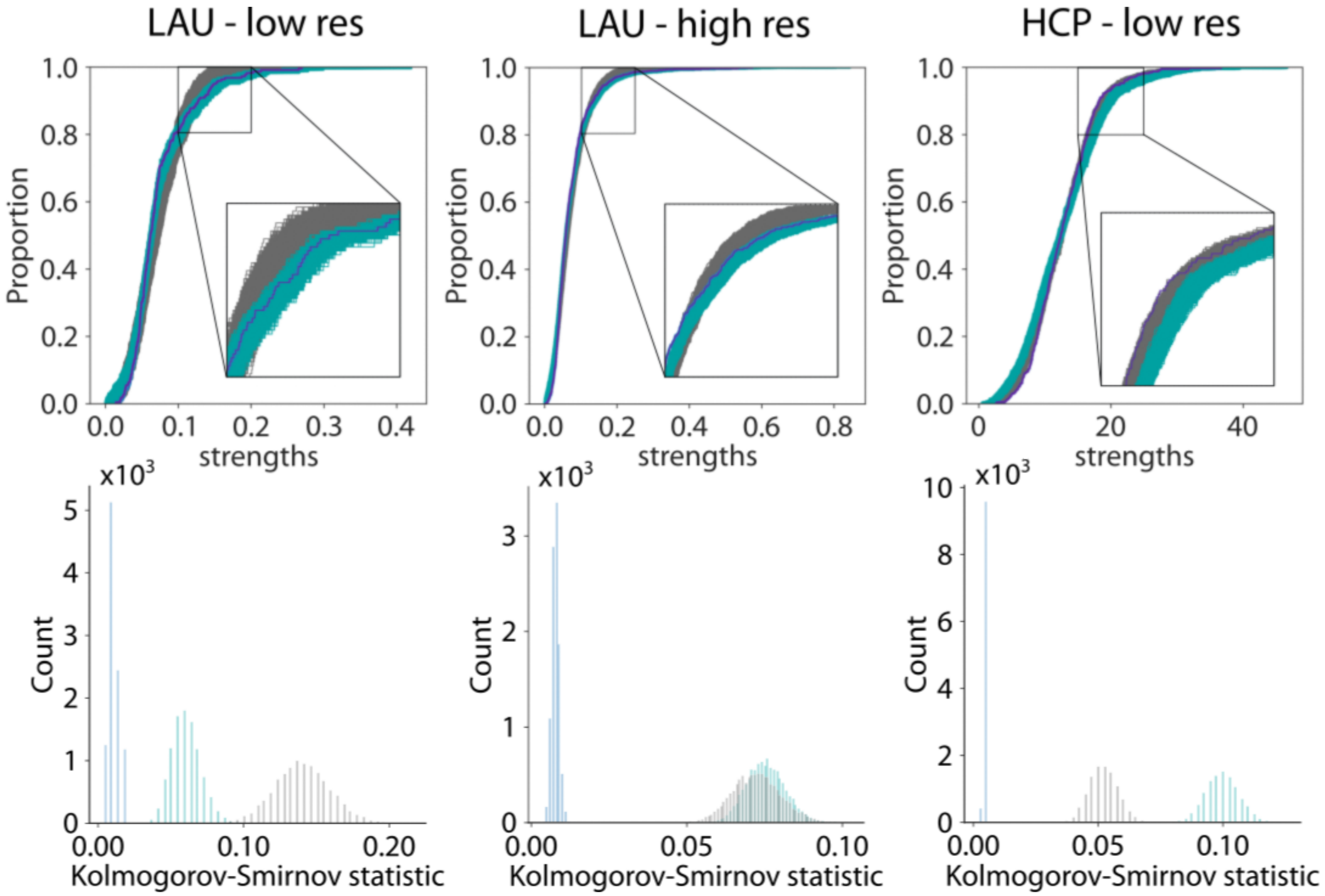

Supplementary Figure S8 **Benchmarking strength distribution preservation** | Strength cumulative distribution functions (top) and histograms representing Kolmogorov–Smirnov statistics obtained by comparing the strength distribution of the empirical network with that of the randomized networks (bottom). Cumulative distribution function curves and histograms are shown in grey for the Maslov–Sneppen algorithm, teal for the Rubinov–Sporns algorithm, and blue for the simulated annealing algorithm. The original cumulative distribution function is depicted in indigo and almost perfectly overlays all 10 000 cumulative distribution functions obtained via simulated annealing, effectively hiding them.

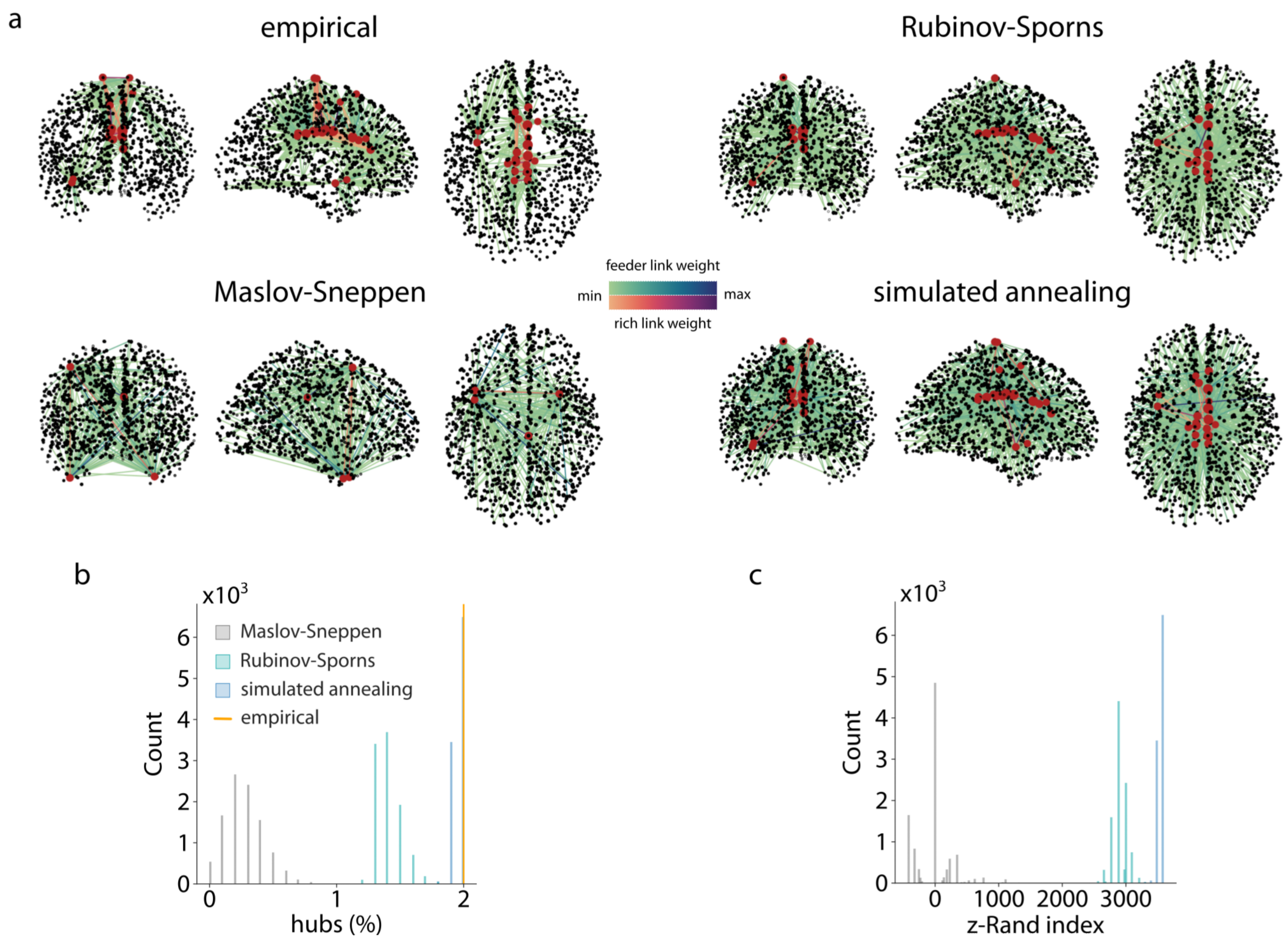

Supplementary Figure S9 **Hubs** | (a) Brain plots representing hubs (red points) identified in the high resolution Lausanne empirical network (top left) and example Maslov–Sneppen (bottom left), Rubinov–Sporns (top right) and simulated annealing (bottom right) null networks. Feeder links (connections between hubs and non-hubs) are colored by weight based on the green-blue colormap, whereas rich links (connections between hubs) are colored by weight based on the red-indigo colormap. Displaying rich/feeder links shows that the network is rewired by all the algorithms, but the hubs are only preserved by the strength-preserving null models (Rubinov–Sporns and simulated annealing). (b) Histogram representing percentage of hubs in the empirical (yellow line) and across null networks (Maslov–Sneppen in grey, Rubinov–Sporns in teal, and simulated annealing in blue). (c) Histogram representing z-scored Rand indices between the empirical and the null hub assignments.

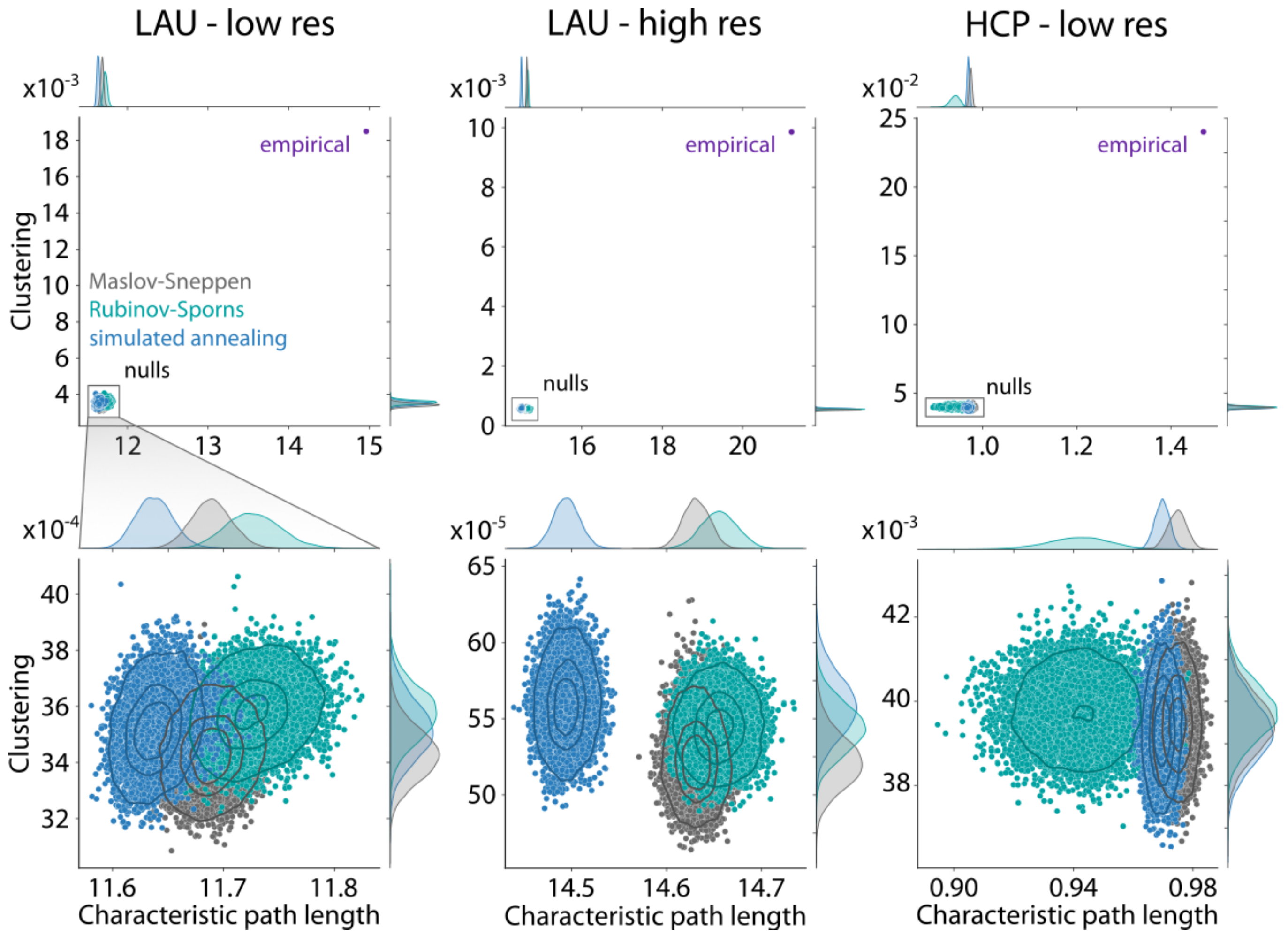

Supplementary Figure S10 **Morphospaces of null network ensembles** | (a) Morphospaces spanned by characteristic path length and clustering. Marginal distribution histograms are shown on the top and right axes. Data points corresponding to randomized null networks generated by the simulated annealing algorithm appear in blue; those resulting from the Rubinov–Sporns algorithm appear in teal; and Maslov–Sneppen rewired networks are shown in grey. The empirical group-consensus structural network is depicted in indigo. The bottom panel consists in a zoomed-in view of the clusters of randomized networks appearing in the top panel. Contour levels are drawn using a Gaussian kernel density estimate and delineate iso-proportions of the density.

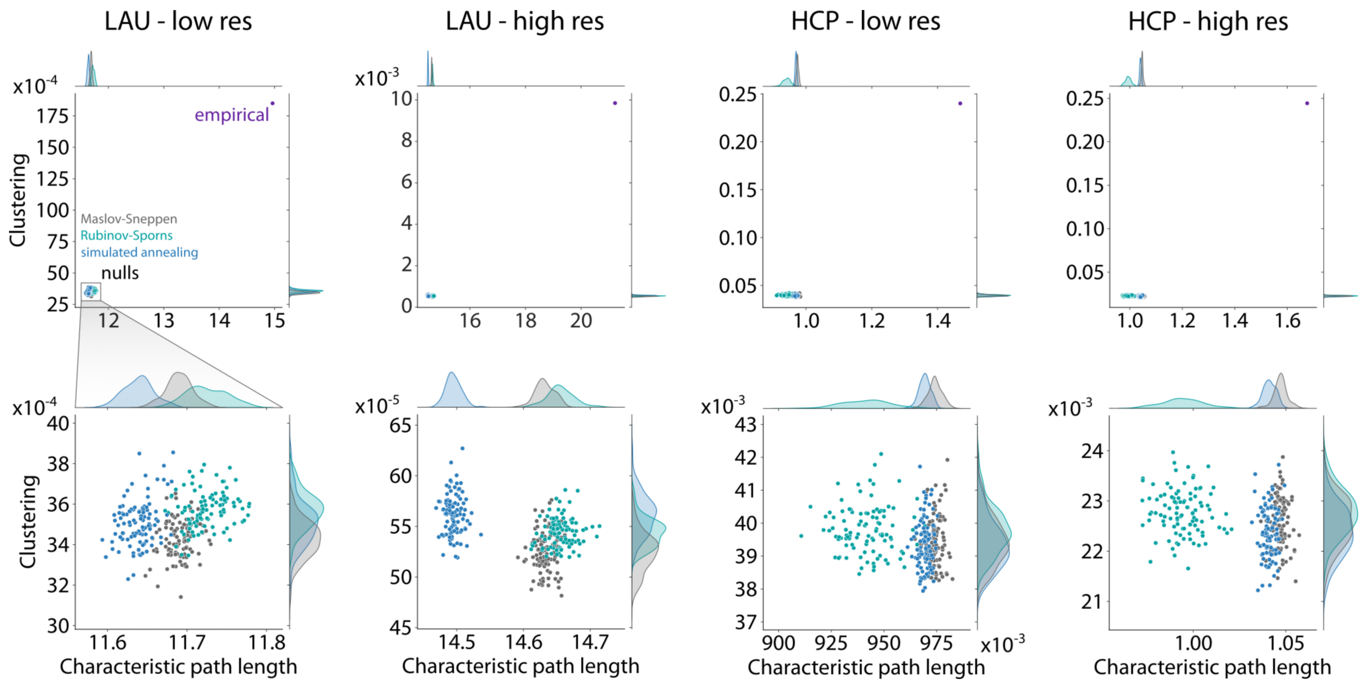

Supplementary Figure S11 **Morphospaces - 100 nulls** | Morphospaces spanned by characteristic path length and clustering for a subset of 100 nulls. Marginal distribution histograms are shown on the top and right axes. Data points corresponding to randomized null networks generated by the simulated annealing algorithm appear in blue; those resulting from the Rubinov–Sporns algorithm appear in teal; and Maslov–Sneppen rewired networks are shown in grey. The empirical group-consensus structural networks are depicted in indigo. The bottom row consists in a zoomed-in view of the clusters of randomized networks appearing in the top row.

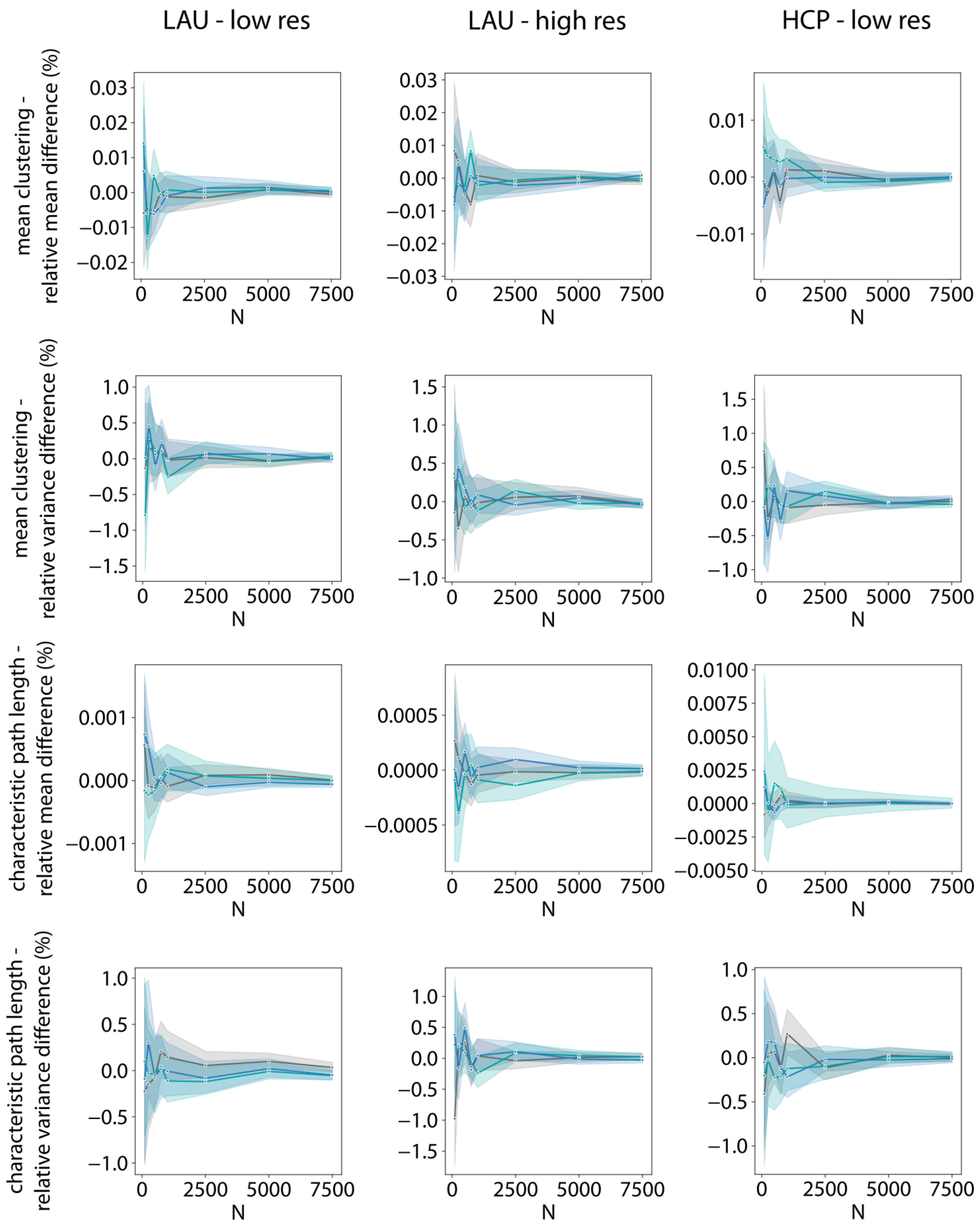

Supplementary Figure S12 **Morphospace scaling behavior** | Trajectories of relative difference in mean clustering (first row), clustering variance (second row), mean characteristic path length (third row) and characteristic path length variance (fourth row) between the full null population ($N = 10\,000$) and subsamples of increasing size ($n \in \{100, 250, 500, 750, 1000, 2500, 5000, 7500\}$). Colored lines and shaded bands represent mean and 95% bootstrapped confidence interval (1000 samples).

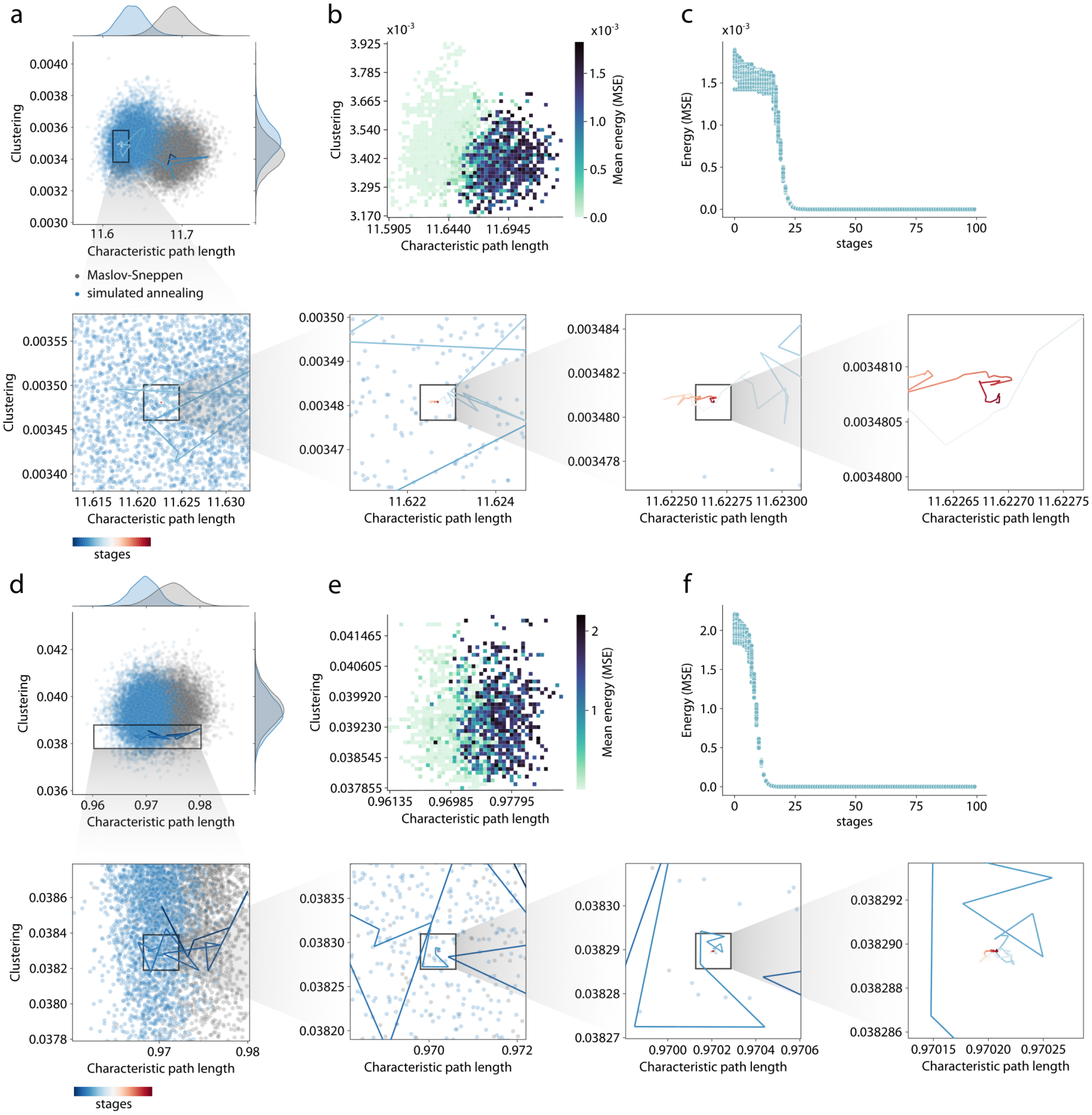

Supplementary Figure S13 **Morphospace trajectories** | (a,d) Example annealing trajectory through a morphospace spanned by characteristic path length and clustering for the low-resolution Lausanne (a) and HCP (d) networks. Marginal distribution histograms are shown on the top and right axes. Data points corresponding to randomized null networks generated by the simulated annealing algorithm appear in blue and Maslov–Sneppen rewired networks are shown in grey. The trajectory is represented by a line colored from blue (early stages) to red (late stages). (b,e) Morphospace colored by mean energy aggregating 100 null network stages per annealing schedule across 100 nulls. (c,f) Optimization trajectories (energy as a function of annealing stage) for 100 null networks.

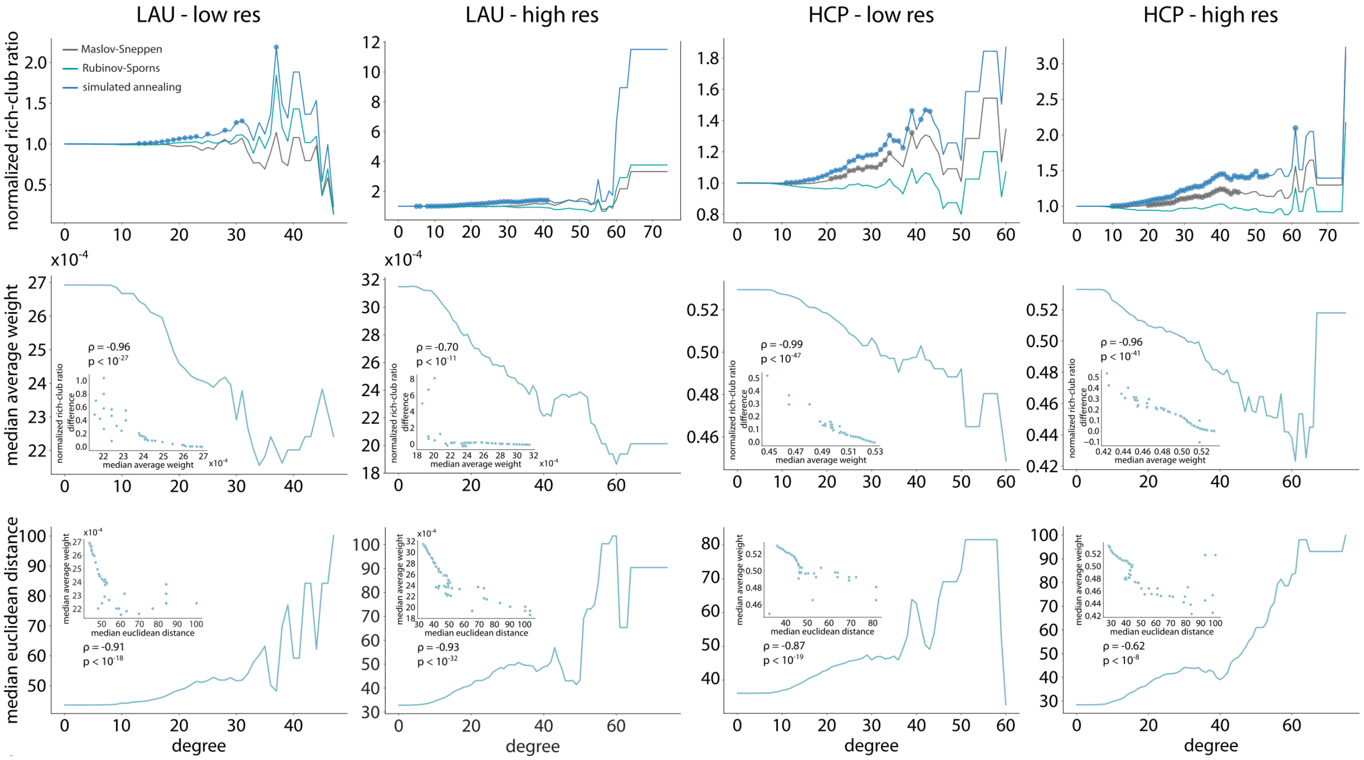

Supplementary Figure S14 **The weighted rich-club phenomenon** | Top: Normalized rich-club ratio as a function of the degree threshold used to define rich nodes. Lines are colored by the null algorithm used (Maslov–Sneppen in grey, Rubinov–Sporns in teal, and simulated annealing in blue). Colored points indicate significance at the Bonferroni-corrected threshold of $p < 0.05$ (one-sided). Middle: Median average weight of the rich nodes as a function of the degree threshold used to define them. Inset: Relationship between the normalized rich-club ratio difference (simulated annealing-derived - Maslov–Sneppen-derived) and the median average weight of the rich nodes. Spearman correlation coefficient and the resulting p-value are indicated above. Bottom: Median Euclidean distance of rich links as a function of the degree threshold used to define them. Inset: Relationship between the normalized rich-club ratio difference (simulated annealing-derived - Maslov–Sneppen-derived) and the median Euclidean distance of rich links. Spearman correlation coefficient and the resulting p-value are indicated below.

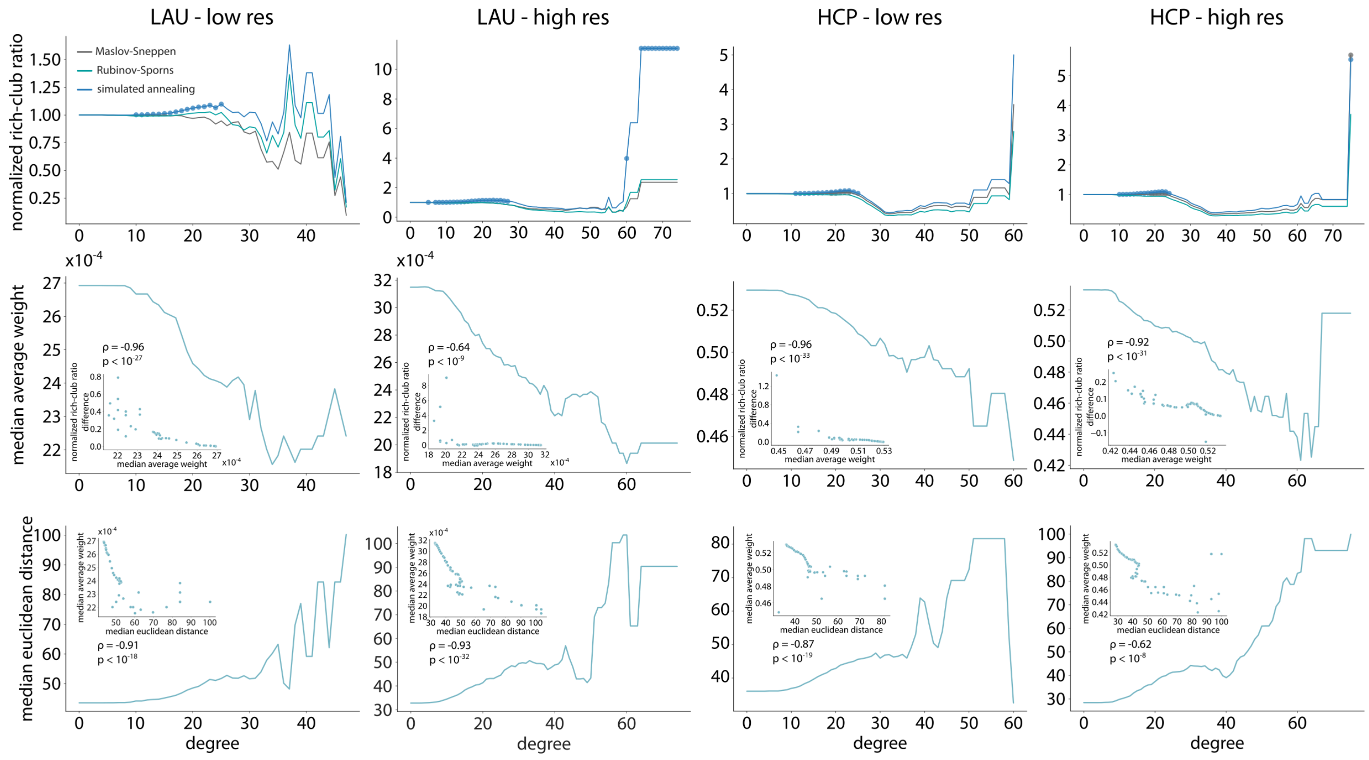

Supplementary Figure S15 **The weighted rich-club phenomenon - alternative coefficient** | Top: Normalized rich-club ratio as a function of the degree threshold used to define rich nodes. Lines are colored by the null algorithm used (Maslov–Sneppen in grey, Rubinov–Sporns in teal, and simulated annealing in blue). Colored points indicate significance at the Bonferroni-corrected threshold of $p < 0.05$ (one-sided). Middle: Median average weight of the rich nodes as a function of the degree threshold used to define them. Inset: Relationship between the normalized rich-club ratio difference (simulated annealing-derived - Maslov–Sneppen-derived) and the median average weight of the rich nodes. Spearman correlation coefficient and the resulting p-value are indicated above. Bottom: Median Euclidean distance of rich links as a function of the degree threshold used to define them. Inset: Relationship between the normalized rich-club ratio difference (simulated annealing-derived - Maslov–Sneppen-derived) and the median Euclidean distance of rich links. Spearman correlation coefficient and the resulting p-value are indicated below.

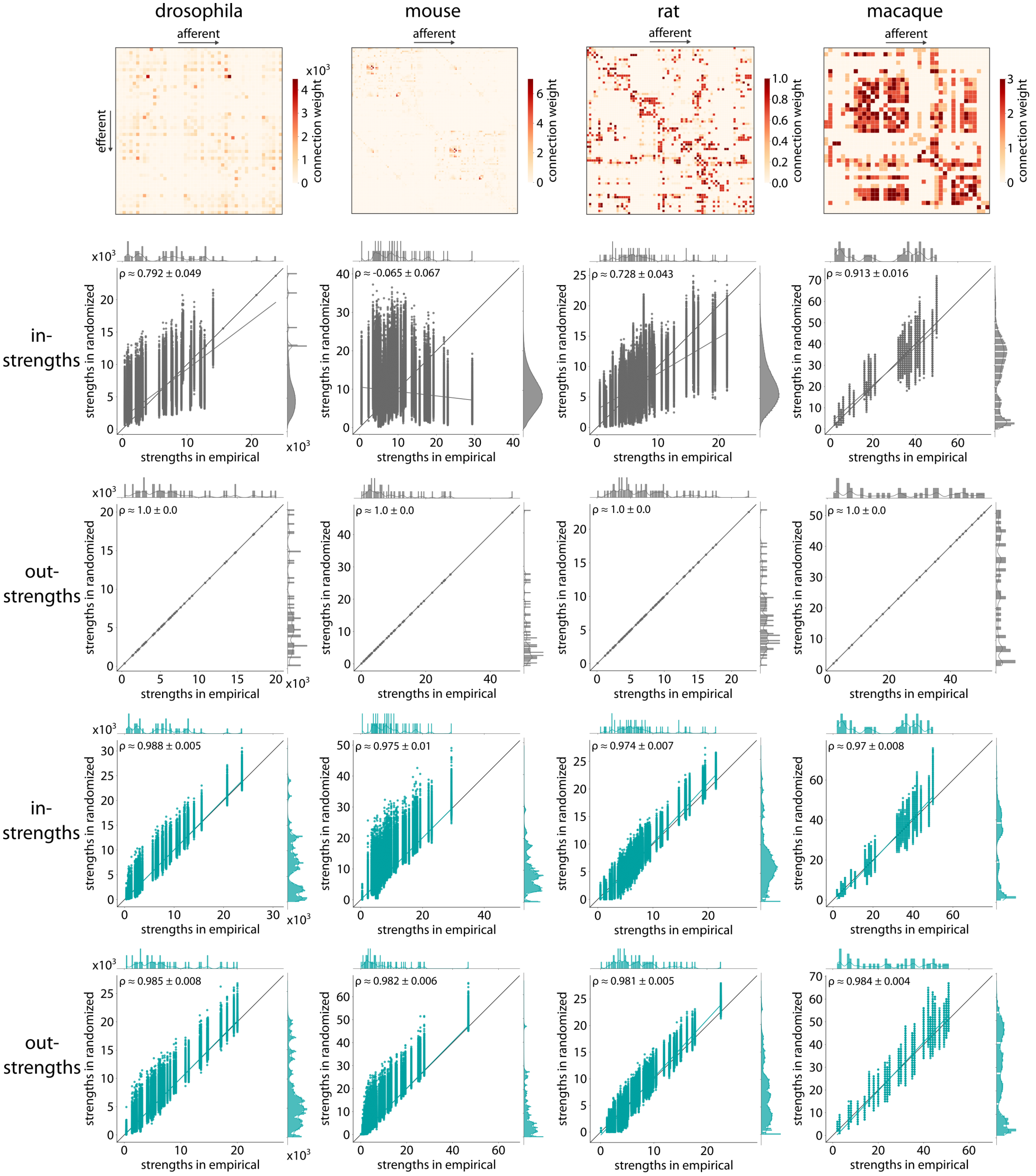

Supplementary Figure S16 **Strength-preserving randomization in directed networks - Maslov–Sneppen and Rubinov–Sporns** | Top: Wiring diagrams for the drosophila, mouse, rat, and macaque connectomes (from left to right). Bottom: Scatter plots of strengths of the empirical (abscissa) and randomized (ordinate) networks for all 10 000 null networks, where each point represents a brain region. In-strengths and out-strengths are considered separately. Note that the Maslov–Sneppen algorithm provides a biased reconstruction of the in-strength sequence, systematically overestimating low strengths and underestimating high strengths. Marginal distribution histograms are shown on the top and right axes. Mean and standard deviation across 10 000 Spearman rank-order correlation coefficients are provided as insets. Data points and histograms appear in grey for the Maslov–Sneppen algorithm and teal for the Rubinov–Sporns algorithm. Linear regression lines (colored) are computed over the whole ensemble for visualization purposes. The identity line (black) is provided as reference.

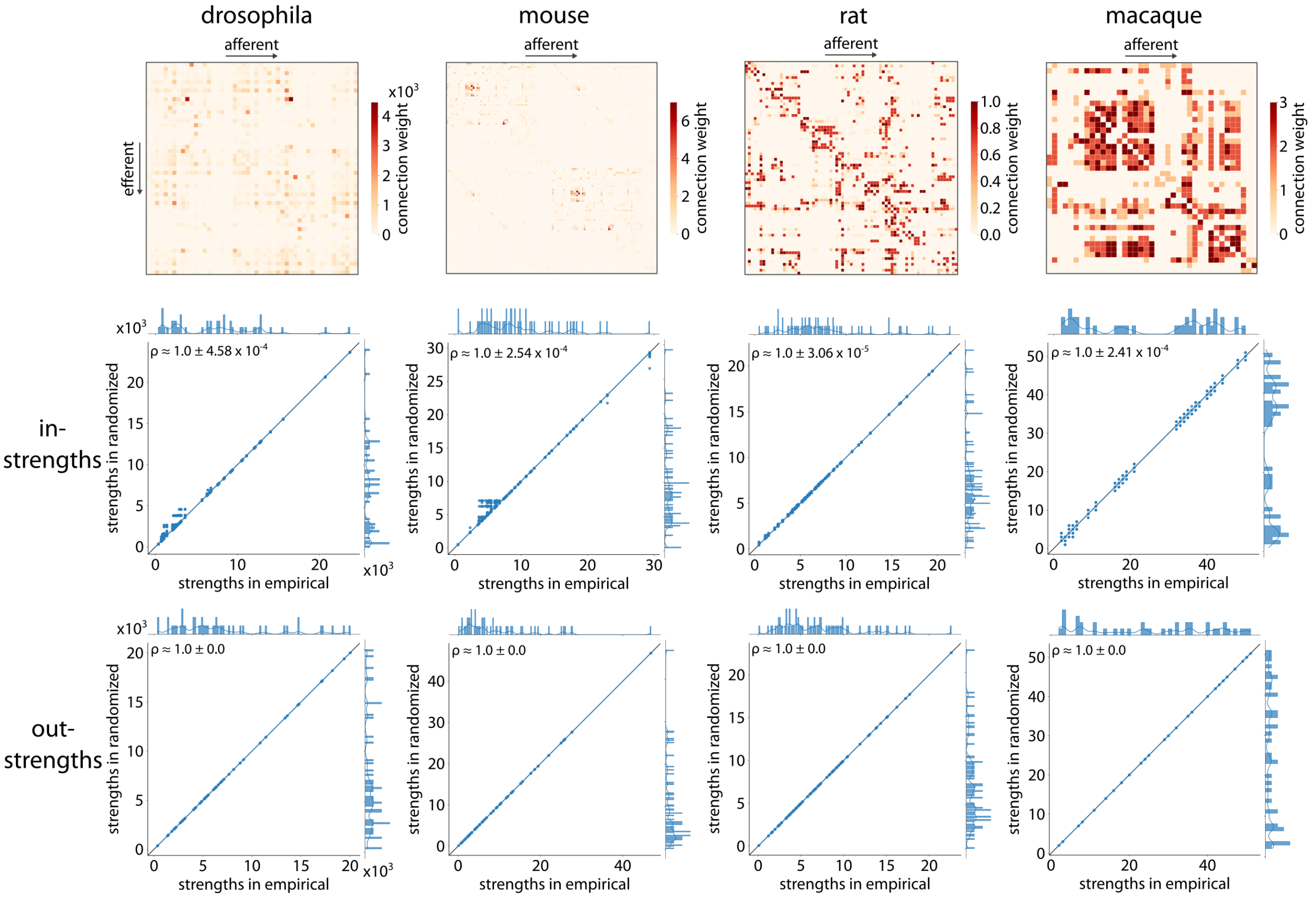

Supplementary Figure S17 **Strength-preserving randomization in directed networks - alternative simulated annealing algorithm** | Top: Wiring diagrams for the drosophila, mouse, rat, and macaque connectomes (from left to right). Bottom: Scatter plots of strengths of the empirical (abscissa) and simulated annealing-derived networks (ordinate) for all 10 000 nulls, where each point represents a brain region. In-strengths and out-strengths are considered separately. Marginal distribution histograms are shown on the top and right axes. Mean and standard deviation across 10 000 Spearman rank-order correlation coefficients are provided as insets. Linear regression lines (blue) are computed over the whole ensemble for visualization purposes. The identity line (black) is provided as reference.

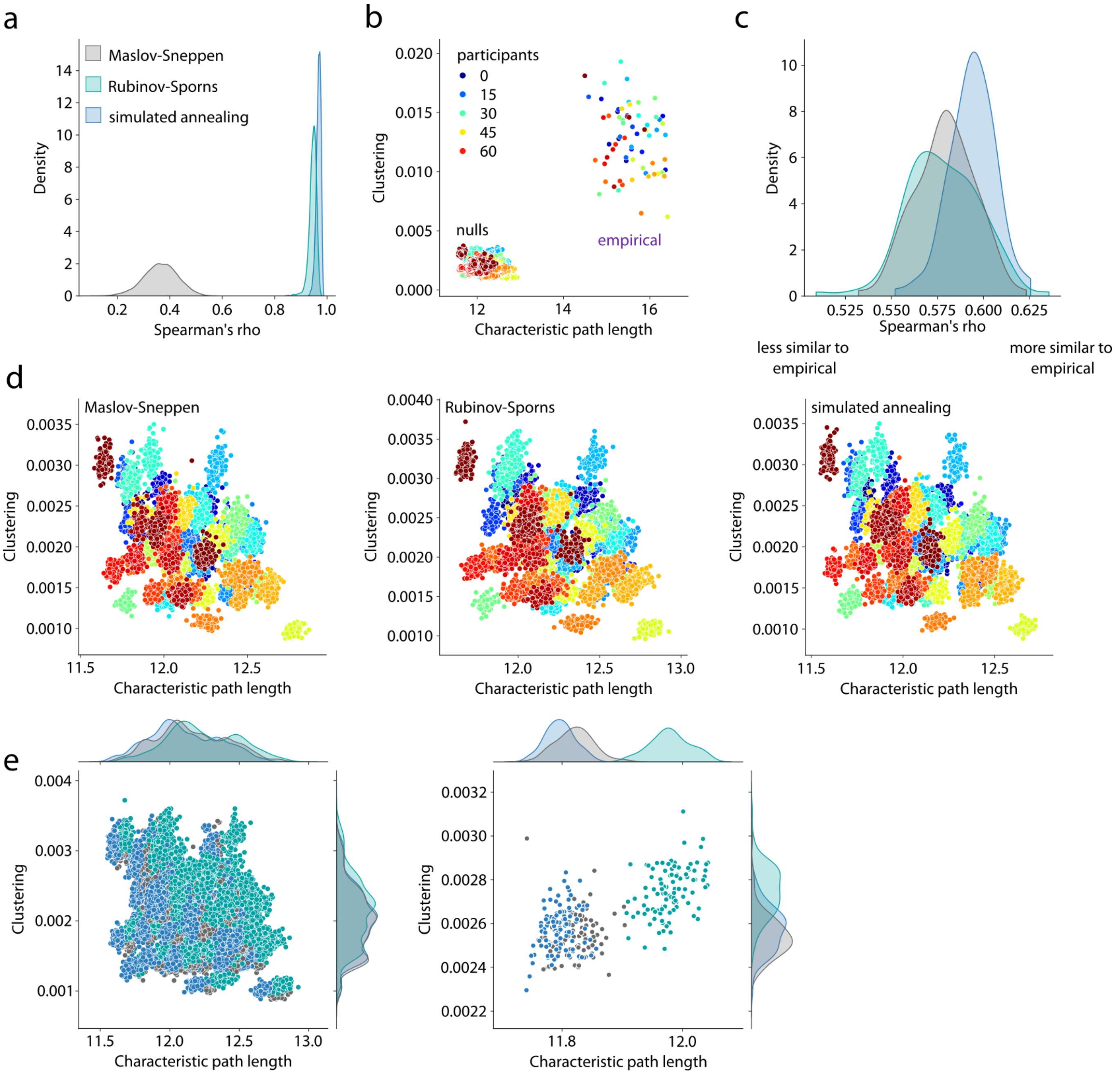

Supplementary Figure S18 **Strength-preserving randomization in individual networks** | (a) Density plot representing Spearman correlation coefficients between strength sequences in empirical and randomized networks derived using the Maslov–Sneppen algorithm (grey), Rubinov–Sporns algorithm (teal), and simulated annealing algorithm (blue). (b) Morphospace spanned by characteristic path length and clustering in which 69 empirical connectomes and a total of 300 null networks per connectome were embedded. Networks are colored by participant. (c) Density plot representing Spearman correlation coefficients between empirical and null sets of Euclidean distances between participants across 100 nulls for each randomization algorithm. (d) Algorithm-wise morphospaces of null networks colored by participant. (e) Left: All null networks embedded in the morphospace. Right: Example null network morphospace for a single participant. Marginal distribution histograms are shown on the top and right axes. Data points are colored by the randomization algorithm used to generate them.

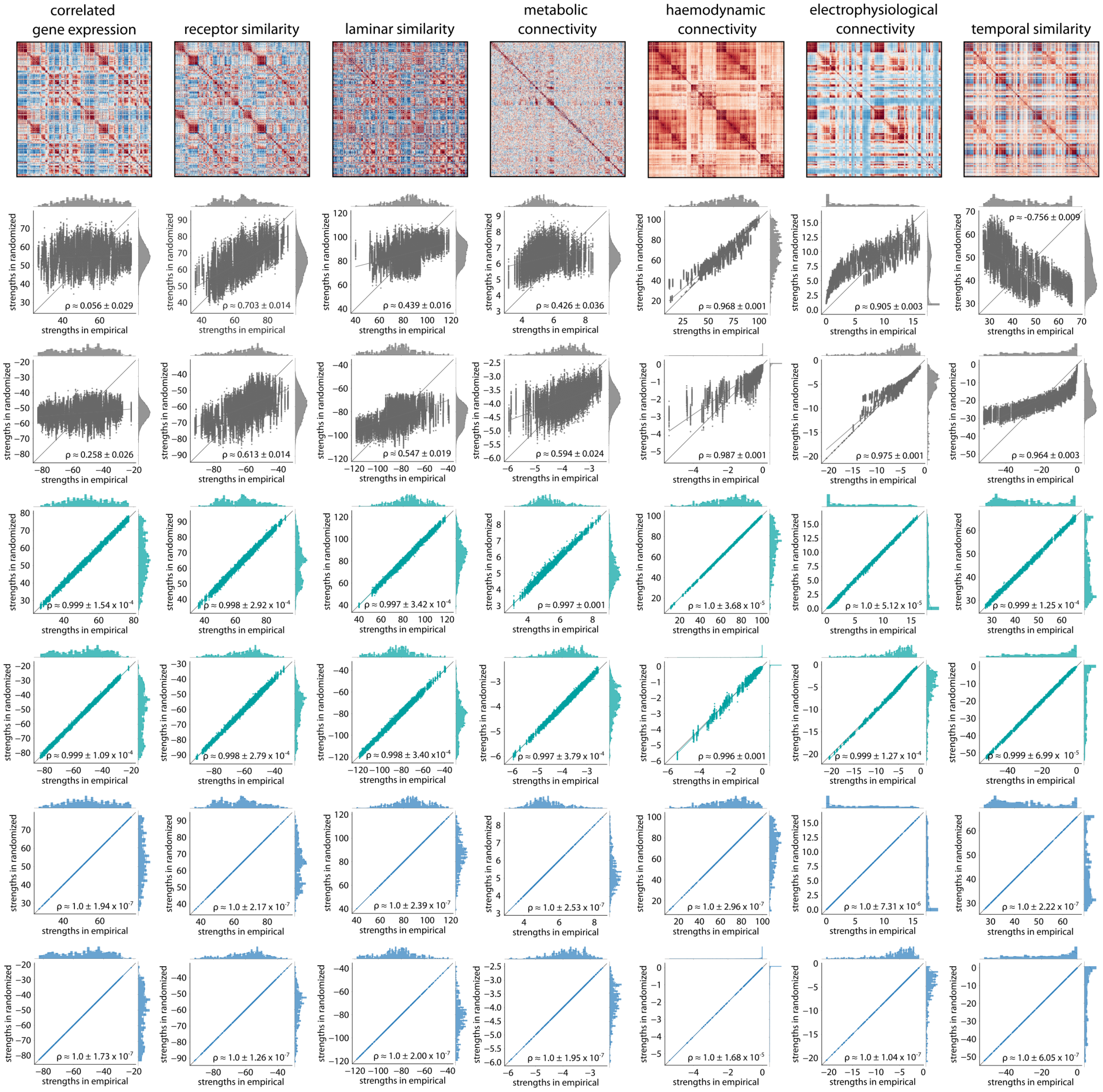

Supplementary Figure S19 **Strength-preserving randomization for signed networks** | Top: Signed brain networks of inter-regional biological similarity. Bottom: Scatter plots of strengths of the empirical (abscissa) and randomized (ordinate) networks for all 100 null networks, where each point represents a brain region. Colors represent the null model used (grey: connection-switching, teal: Rubinov–Sporns, blue: simulated annealing). Positive (top rows) and negative (bottom rows) strengths are considered separately for each algorithm. Marginal distribution histograms are shown on the top and right axes. Mean and standard deviation across 100 Spearman rank-order correlation coefficients are provided as insets. Linear regression lines (colored) are computed over the whole ensemble for visualization purposes. The identity line (black) is provided as reference.

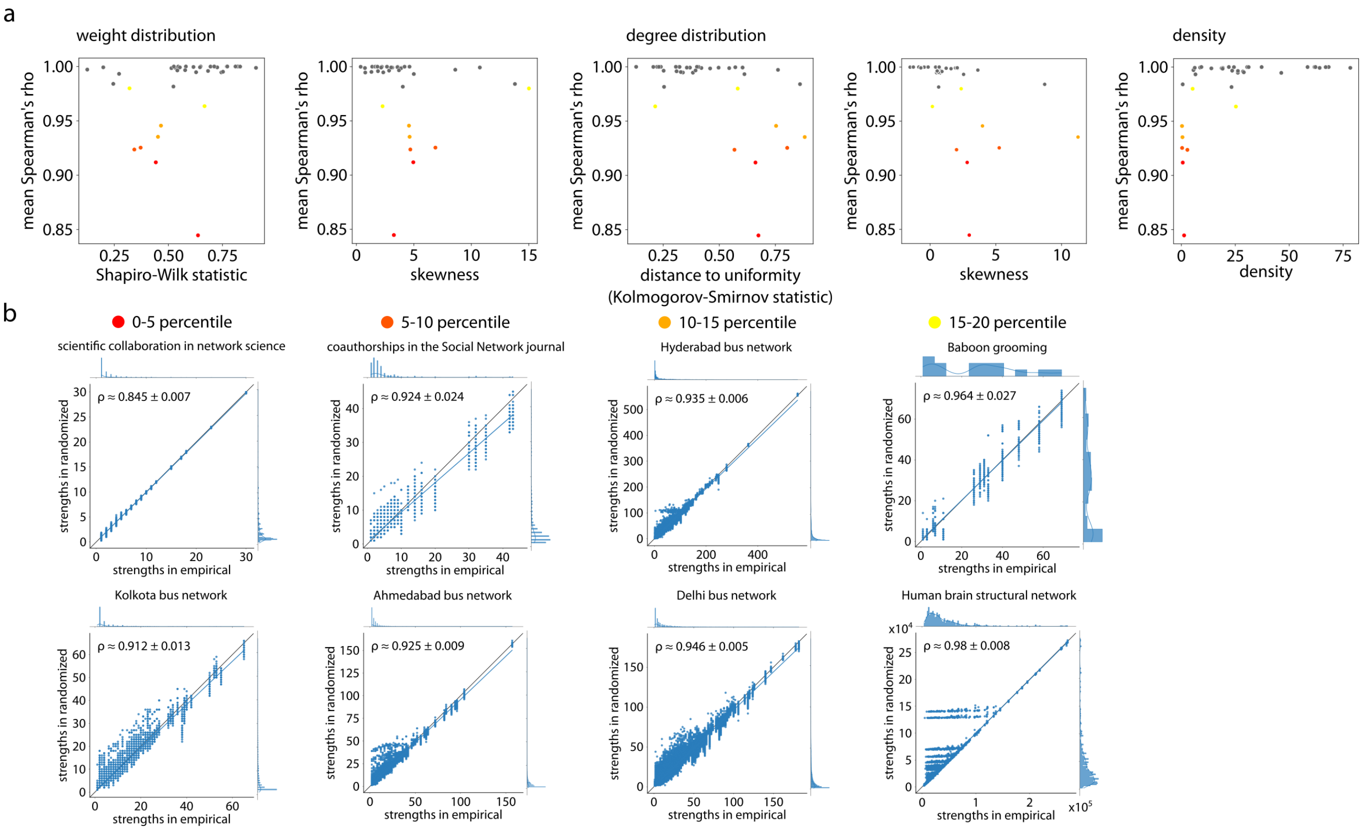

Supplementary Figure S20 **Network determinants of simulated annealing performance** | (a) Mean Spearman's rho for strength reconstruction as a function of the weight distribution normality (Shapiro–Wilk statistic) and skewness, degree distribution distance to uniformity (Kolmogorov–Smirnov statistic) and skewness, and network density (from left to right). Outliers (Mean Spearman's rho below 20th percentile) are colored in groups of 5 percentiles, e.g., points colored in orange in the $10 - 15$ percentile group are networks with strength reconstruction performance contained between the 10th and 15th percentile of the distribution. The rest of the networks are colored in grey. (b) Scatter plots of strengths of the empirical (abscissa) and randomized (ordinate) networks for all 100 null networks, where each point represents a node. Marginal distribution histograms are shown on the top and right axes. Mean and standard deviation across 100 Spearman rank-order correlation coefficients are provided as insets. Scatter plots are grouped in columns of 5 percentiles according to the mean correlation coefficients. Linear regression lines (blue) are computed over the whole ensemble for visualization purposes. The identity line (black) is provided as reference.

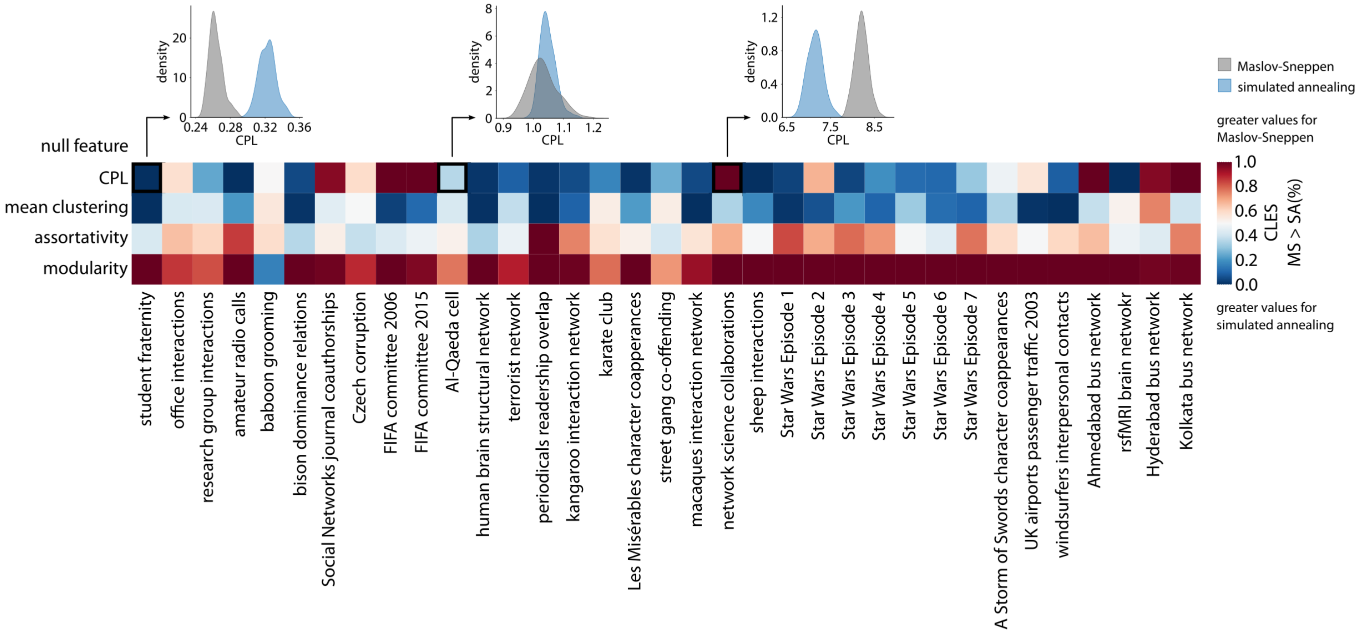

Supplementary Figure S21 **Influence of strength on global network features** | Heatmap of common-language effect sizes (CLES; percentage of values from the degree-preserving null ensemble that are greater than values from the strength-preserving null ensemble) for global network features (rows), namely characteristic path length (CPL), mean clustering, assortativity, and modularity, across diverse real-world networks (columns). Red indicates greater values for the degree-preserving nulls obtained via Maslov–Sneppen rewiring, whereas blue indicates higher values for the strength-preserving nulls obtained via simulated annealing. Example density plots for CPL are shown on top to reflect how CLES captures the overlap between the Maslov–Sneppen (grey) and the simulated annealing (blue) distributions.
