# Supplementary material for: A simulated annealing algorithm for randomizing weighted networks
Source: Nat Comput Sci. 2024 Dec 10;5(1):48–64. doi: 10.1038/s43588-024-00735-z (PMC11774763; doi:10.1038/s43588-024-00735-z)
Supplement: Supplementary file 2 — Reporting Summary [file 43588_2024_735_MOESM2_ESM.pdf]

nature portfolio

Corresponding author(s): Bratislav Misic

Last updated by author(s): Mar 22, 2024

Reporting Summary

Nature Portfolio wishes to improve the reproducibility of the work that we publish. This form provides structure for consistency and transparency in reporting. For further information on Nature Portfolio policies, see our Editorial Policies and the Editorial Policy Checklist.

Statistics

For all statistical analyses, confirm that the following items are present in the figure legend, table legend, main text, or Methods section.

n/a	Confirmed	
☐	☒	The exact sample size (*n*) for each experimental group/condition, given as a discrete number and unit of measurement
☒	☐	A statement on whether measurements were taken from distinct samples or whether the same sample was measured repeatedly
☐	☒	The statistical test(s) used AND whether they are one- or two-sided<br>*Only common tests should be described solely by name; describe more complex techniques in the Methods section.*
☒	☐	A description of all covariates tested
☐	☒	A description of any assumptions or corrections, such as tests of normality and adjustment for multiple comparisons
☐	☒	A full description of the statistical parameters including central tendency (e.g. means) or other basic estimates (e.g. regression coefficient) AND variation (e.g. standard deviation) or associated estimates of uncertainty (e.g. confidence intervals)
☐	☒	For null hypothesis testing, the test statistic (e.g. *F*, *t*, *r*) with confidence intervals, effect sizes, degrees of freedom and *P* value noted<br>*Give P values as exact values whenever suitable.*
☒	☐	For Bayesian analysis, information on the choice of priors and Markov chain Monte Carlo settings
☒	☐	For hierarchical and complex designs, identification of the appropriate level for tests and full reporting of outcomes
☐	☒	Estimates of effect sizes (e.g. Cohen's *d*, Pearson's *r*), indicating how they were calculated

Our web collection on statistics for biologists contains articles on many of the points above.

Software and code

Policy information about availability of computer code

Data collection	For the Lausanne dataset, the FreeSurfer version 5.0.0 open-source package was employed to segment white matter and grey matter from the MPRAGE volumes, whereas tools from the Connectome Mapper 1.2.0 open-source software were used to preprocess DSI data.<br>HCP structural data was processed using MRtrix3 v3.0.0.
Data analysis	All code used for data analysis is available at https://github.com/netneurolab/milisav_strength_nulls.<br>Python v3.9.17 was used for running scripts. A full list of packages used can be found at https://github.com/netneurolab/milisav_strength_nulls/blob/main/code/analysis/milisav_str_nulls_analysis.yml. The core packages include netneurotools v0.2.3 (https://netneurotools.readthedocs.io/en/latest/index.html), bctpy v0.6.1 (https://github.com/aestrivex/bctpy), scipy v1.10.1 (https://docs.scipy.org/doc/scipy/index.html), and pingouin v0.5.3 (https://pingouin-stats.org/build/html/index.html).

For manuscripts utilizing custom algorithms or software that are central to the research but not yet described in published literature, software must be made available to editors and reviewers. We strongly encourage code deposition in a community repository (e.g. GitHub). See the Nature Portfolio guidelines for submitting code & software for further information.

Data

Policy information about availability of data

All manuscripts must include a data availability statement. This statement should provide the following information, where applicable:

- Accession codes, unique identifiers, or web links for publicly available datasets
- A description of any restrictions on data availability
- For clinical datasets or third party data, please ensure that the statement adheres to our policy

Original and intermediate, preprocessed, data used in this work is available at https://github.com/netneurolab/milisav_strength_nulls. The Lausanne dataset is available at https://zenodo.org/records/2872624. The HCP dataset is available at https://db.humanconnectome.org/data/projects/HCP_1200. The drosophila connectome is available at https://doi.org/10.1016/j.cub.2015.03.021. The Allen Mouse Connectivity (connectivity.brain-map.org), Developing Mouse Brain (developingmouse.brain-map.org), and Mouse Brain (mouse.brain-map.org) atlases are made openly available by the Allen Institute for Brain Science. The CoCoMac database is available at http://cocomac.g-node.org. The rat connectome is available at https://bams1.org/connectomes/standard_rat.php. The dataset of real-world complex weighted networks is available at https://figshare.com/s/22c5b72b574351d03edf?file=25762442. Source Data for Figures 2b, 3, 4, and 6 is available with this manuscript. Large Source Data files for Figures 2a and 5 are available at https://doi.org/10.5281/zenodo.13988937.

Human research participants

Policy information about studies involving human research participants and Sex and Gender in Research.

Reporting on sex and gender	Structural connectomes were group consensus networks generated from individual connectomes of participants of both sexes. Gender was not considered in the analyses.
Population characteristics	Lausanne : age 28.8 +/- 8.9 years old, 37% females. HCP: age 28.6 +/- 3.73 years old, 55% females.
Recruitment	Only data from healthy young adults were used in the analyses.
Ethics oversight	Lausanne: Informed written consent was provided by all participants in accordance with institutional guidelines and the protocol was approved by the Ethics Committee of Clinical Research of the Faculty of Biology and Medicine, University of Lausanne, Switzerland.<br>HCP: Informed written consent was provided by all participants and the protocol was approved by the Washington University Institutional Review Board.

Note that full information on the approval of the study protocol must also be provided in the manuscript.

Field-specific reporting

Please select the one below that is the best fit for your research. If you are not sure, read the appropriate sections before making your selection.

☒ Life sciences ☐ Behavioural & social sciences ☐ Ecological, evolutionary & environmental sciences

For a reference copy of the document with all sections, see nature.com/documents/nr-reporting-summary-flat.pdf

Life sciences study design

All studies must disclose on these points even when the disclosure is negative.

Sample size	Sample sizes of individual datasets were not chosen as only open-source data was used. Group consensus networks were formed from individual participant data and statistical analyses were performed on ensembles of null networks derived from empirical networks. Most analyses were performed on ensembles of 10000 nulls, which reflects a typical usage scenario given the associated computational cost. Note also that we find that even much smaller samples (as low as 100 nulls) are sufficient to robustly approximate the null distributions of global network features.
Data exclusions	No data was excluded.
Replication	Network randomization algorithms were tested in two publicly available diffusion-weighted MRI datasets, acquired using different protocols (diffusion spectrum imaging; DSI, and high angular resolution diffusion imaging; HARDI), parcellations (anatomical and functional) and parcellation resolutions (low and high in each dataset), as well as in individual structural connectivity networks from the Lausanne dataset, directed animal connectomes, and real-world complex networks from the Index of Complex Networks (ICON). Across all replication scenarios from 6 independent datasets, we consistently find that the simulated annealing algorithm exhibits better performance in reconstructing the empirical network’s strength distribution and sequence.
Randomization	Randomization was not performed because participants were not placed into experimental groups.
Blinding	Blinding is not relevant to this study because participants were not placed into experimental groups.

Reporting for specific materials, systems and methods

We require information from authors about some types of materials, experimental systems and methods used in many studies. Here, indicate whether each material, system or method listed is relevant to your study. If you are not sure if a list item applies to your research, read the appropriate section before selecting a response.

Materials & experimental systems

n/a	Involved in the study
☒	☐ Antibodies
☒	☐ Eukaryotic cell lines
☒	☐ Palaeontology and archaeology
☐	☒ Animals and other organisms
☒	☐ Clinical data
☒	☐ Dual use research of concern

Methods

n/a	Involved in the study
☒	☐ ChIP-seq
☒	☐ Flow cytometry
☐	☒ MRI-based neuroimaging

Animals and other research organisms

Policy information about studies involving animals; ARRIVE guidelines recommended for reporting animal research, and Sex and Gender in Research

Laboratory animals	For full information on laboratory animals, please refer to the original articles:<br>Drosophila: https://doi.org/10.1016/j.cub.2015.03.021<br>Mouse: https://doi.org/10.1073/pnas.1420315112<br>Rat: https://doi.org/10.1073/pnas.1504394112<br>Macaque: https://doi.org/10.1523/JNEUROSCI.0752-14.2014
Wild animals	This study did not involve wild animals.
Reporting on sex	The drosophila connectome was reconstructed using projection neuron images of the female drosophila brain.<br>For full information on laboratory animals, please refer to the original articles:<br>Drosophila: https://doi.org/10.1016/j.cub.2015.03.021<br>Mouse: https://doi.org/10.1073/pnas.1420315112<br>Rat: https://doi.org/10.1073/pnas.1504394112<br>Macaque: https://doi.org/10.1523/JNEUROSCI.0752-14.2014
Field-collected samples	This study did not involve samples collected from the field.
Ethics oversight	For full information on laboratory animals, please refer to the original articles:<br>Drosophila: https://doi.org/10.1016/j.cub.2015.03.021<br>Mouse: https://doi.org/10.1073/pnas.1420315112<br>Rat: https://doi.org/10.1073/pnas.1504394112<br>Macaque: https://doi.org/10.1523/JNEUROSCI.0752-14.2014

Note that full information on the approval of the study protocol must also be provided in the manuscript.

Magnetic resonance imaging

Experimental design

Design type	Structural MRI and diffusion-weighted MRI
Design specifications	No trial structure.
Behavioral performance measures	No behavioral measures were acquired.

Acquisition

Imaging type(s)	Structural MRI and diffusion-weighted MRI
Field strength	3T
Sequence & imaging parameters	Lausanne: (1) a magnetization-prepared rapid acquisition gradient echo (MPRAGE) sequence sensitive to white/gray matter contrast (1 mm in-plane resolution, 1.2 mm slice thickness) and (2) a diffusion spectrum imaging (DSI) sequence (128 diffusion-weighted volumes and a single b0 volume, maximum b-value 8000 s/mm2, 2.2 × 2.2 × 3.0 mm voxel size).

HCP: (1) a magnetization-prepared rapid acquisition gradient echo (MPRAGE) sequence (TR = 2400 ms, TE = 2.14 ms, FOV = 224 mm ×224 mm, voxel size = 0.7 mm3, 256 slices) and (2) a spin-echo echo-planar imaging (EPI) sequence (TR = 5520 ms, TE = 89.5 ms, FOV = 210 mm ×180 mm, voxel size = 1.25 mm3, b-value = 1000, 2000, and 3000 s/mm2, 270 diffusion directions, 18 b0 volumes)

Area of acquisition: Whole brain

Diffusion MRI: ☒ Used ☐ Not used

Parameters: Lausanne: diffusion spectrum imaging (DSI) sequence (128 diffusion-weighted volumes and a single b0 volume, maximum b-value 8000 s/mm2, 2.2 × 2.2 × 3.0 mm voxel size)
HCP: spin-echo echo-planar imaging (EPI) sequence (TR = 5520 ms, TE = 89.5 ms, FOV = 210 mm ×180 mm, voxel size = 1.25 mm3, b-value = three different shells, i.e., 1000, 2000, and 3000 s/mm2, 270 diffusion directions, 18 b0 volumes)

Preprocessing

Preprocessing software: Lausanne: The FreeSurfer version 5.0.0 open-source package was employed to segment white matter and grey matter from the MPRAGE volumes, whereas the Connectome Mapper pipeline was used to preprocess DSI data. For each white matter voxel, 32 streamline propagations were initiated per diffusion direction. For further details about data preprocessing, please refer to https://zenodo.org/records/2872624.
HCP: The HCP minimal preprocessing pipelines were applied to the MRI data and streamline tractography tools from the MRtrix3 open-source software were used to reconstruct structural connectivity networks in individual participants from diffusion-weighted MRI (dMRI) data. The MPRAGE volume was segmented into white matter, grey matter, and cerebrospinal fluid to perform anatomically constrained tractography. Grey matter was divided according to the 400 and 800 cortical regions resolutions of the Schaefer functional parcellation. The multi-shell multi-tissue constrained spherical deconvolution algorithm from MRtrix3 was used to generate fiber orientation distributions. The tractogram was initialized with 40 million streamlines and constrained with a maximum tract length of 250 and a fractional anisotropy cutoff of 0.06. A spherical deconvolution-informed filtering procedure (SIFT2) was then applied following Smith et al. (2015) to estimate streamline-wise cross-section multipliers. For further details about data preprocessing, please refer to https://doi.org/10.1016/j.neuroimage.2020.117429.

Normalization: Lausanne: For further details about data preprocessing, please refer to https://zenodo.org/records/2872624.
HCP: For further details about data preprocessing, please refer to https://doi.org/10.1016/j.neuroimage.2020.117429.

Normalization template: Lausanne: For further details about data preprocessing, please refer to https://zenodo.org/records/2872624.
HCP: For further details about data preprocessing, please refer to https://doi.org/10.1016/j.neuroimage.2020.117429.

Noise and artifact removal: Lausanne: For further details about data preprocessing, please refer to https://zenodo.org/records/2872624.
HCP: For further details about data preprocessing, please refer to https://doi.org/10.1016/j.neuroimage.2020.117429.

Volume censoring: Lausanne: For further details about data preprocessing, please refer to https://zenodo.org/records/2872624.
HCP: For further details about data preprocessing, please refer to https://doi.org/10.1016/j.neuroimage.2020.117429.

Statistical modeling & inference

Model type and settings: Network randomization models and graph analysis was applied to group consensus structural networks.

Effect(s) tested: We tested differences in strength preservation and graph measures across null network models using Wilcoxon–Mann–Whitney two-sample rank-sum tests.

Specify type of analysis: ☒ Whole brain ☐ ROI-based ☐ Both

Statistic type for inference (See Eklund et al. 2016): N/A

Correction: N/A

Models & analysis

n/a	Involved in the study
☒	☐ Functional and/or effective connectivity
☐	☒ Graph analysis
☒	☐ Multivariate modeling or predictive analysis

Graph analysis: Graph analysis was applied on weighted group consensus structural networks. Structural connectivity was defined as the streamline density between node pairs, i.e., the number of streamlines between two regions normalized by the mean length of the streamlines and the mean surface area of the regions. The graph measures under consideration included degree, strength, the global weighted clustering coefficient, weighted characteristic path length, and the weighted rich-club coefficient.
